# An Evolutionarily Conserved Mesodermal Enhancer in Vertebrate Zic3

**DOI:** 10.1038/s41598-018-33235-y

**Published:** 2018-10-08

**Authors:** Yuri S. Odaka, Takahide Tohmonda, Atsushi Toyoda, Jun Aruga

**Affiliations:** 1 Laboratory for Behavioral and Developmental Disorders, RIKEN Brain Science Institute, Wako-shi, Saitama, 351-0198 Japan; 2 Comparative Genomics Laboratory, Center for Information Biology, National Institute of Genetics, Mishima, Shizuoka, 411-8540 Japan; 3 Department of Medical Pharmacology, Nagasaki University Institute of Biomedical Sciences, Nagasaki, Nagasaki, 852-8523 Japan

## Abstract

*Zic3* encodes a zinc finger protein essential for the development of meso-ectodermal tissues. In mammals, Zic3 has important roles in the development of neural tube, axial skeletons, left-right body axis, and in maintaining pluripotency of ES cells. Here we characterized cis-regulatory elements required for *Zic3* expression. Enhancer activities of human-chicken-conserved noncoding sequences around *Zic1* and *Zic3* were screened using chick whole-embryo electroporation. We identified enhancers for meso-ectodermal tissues. Among them, a mesodermal enhancer (Zic3-ME) in distant 3′ flanking showed robust enhancement of reporter gene expression in the mesodermal tissue of chicken and mouse embryos, and was required for mesodermal Zic3 expression in mice. Zic3-ME minimal core region is included in the DNase hypersensitive region of ES cells, mesoderm, and neural progenitors, and was bound by T (Brachyury), Eomes, Lef1, Nanog, Oct4, and Zic2. Zic3-ME is derived from an ancestral sequence shared with a sequence encoding a mitochondrial enzyme. These results indicate that Zic3-ME is an integrated cis-regulatory element essential for the proper expression of *Zic3* in vertebrates, serving as a hub for a gene regulatory network including *Zic3*.

## Introduction

Zic family of zinc finger proteins is a versatile toolkit for metazoan development^1^. They are involved in the regulation of gene expression during cell-fate decision, regulation of cell proliferation and physiology^2–4^. Vertebrates possess five Zic-related genes (*Zic1-5*) except teleost fishes, which possess six *Zic* genes^1^, among which *Zic1-3* are proposed to be generated in a vertebrate ancestor^5^, and play essential roles in the development of the nervous system and mesodermal derivatives, including axial skeleton and somites.

*Zic3* is required for the maintenance of pluripotency in embryonic stem (ES) cells^6^, neuroectoderm and mesoderm differentiation, and determination of left-right axis of the internal organs^7^. In humans, *Zic3* is a causal gene for heterotaxy and VACTERL association^8–14^. The other vertebrate-specific Zic subtypes, *Zic1* and *Zic2*, contribute differentially to vertebrate development. *Zic1* plays essential roles in neural development^2^. It enhances neural differentiation and its deficiency causes the cerebellar dysgenesis in humans and mice^15^; whereas, *Zic2* is required for medial forebrain development in humans and mice^16^.

The apparently different roles of the *Zic* genes in vertebrate development may be partly explained by their differential expression profiles^17–19^ because similar protein functions are revealed in some comparative studies^20,21^. As a basis for the spatiotemporally defined *Zic* expression profiles, several upstream signaling and trans-acting factors have been described for *Zic1* and *Zic3*. For instance, suppression of the BMP signal is a common upstream factor for the upregulation of *Xenopus Zic1* and *Zic3* during neuroectodermal differentiation^22,23^. In addition, Sonic Hedgehog acts as an inhibitory factor for mouse *Zic1* expression in the ventral side^24^. At later stages, the involvement of Meis1/Pbx induces *Zic3* expression in the generation of neural crest cells^25^. Moreover, Brachyury upregulates the mesodermal expression of *Xenopus Zic3*^26^.

Several studies have addressed the role of cis-regulatory elements, along with the associated signaling and transcription factors (TFs), in the regulation of *Zic* gene expression. Examples of these cis-regulatory elements include- (1) The 5′ flanking region of mouse *Zic1* that controls the dorsal spinal cord expression^27^; (2) The BMP inhibitor-responsive promoter region in *Xenopus Zic1*^28^. (3) The midbrain-hindbrain enhancer for zebrafish (*Danio rerio*) *Zic2* and *Zic5*^29^; (4) The neural plate border enhancer for zebrafish *Zic3* and *Zic6*^30^, and (5) The somite-mesodermal enhancer for *Zic1* and *Zic4*^31^. However, these regulatory elements still do not fully explain the *Zic* expression profiles, and none of these elements has been shown to be necessary for *Zic* expression during development.

To understand the mechanism underlying the regulation of *Zic* gene expression, we analyzed the cis-regulatory elements required for the expression of vertebrate *Zic1* and *Zic3*, and characterized a developmentally critical *Zic3* mesodermal enhancer in terms of its activity in chicken and mouse embryos, potential trans-acting factors, and its evolutionary history.

## Results

### *Zic1* and *Zic3* expression in early chick development

To identify enhancers critical for embryonic *Zic* expression, we used chicken embryos as an experimental system. In chicken embryos, expression of *Zic1–Zic4* has been described in neuroectoderm, paraxial mesenchyme, brain, spinal cord, neural crest, inner ear, and limb buds^24,32–34^. We chose *Zic1* and *Zic3*, whose regulatory signaling cascades and cis-elements are partly known, as targets of analysis.

**Figure 1:**
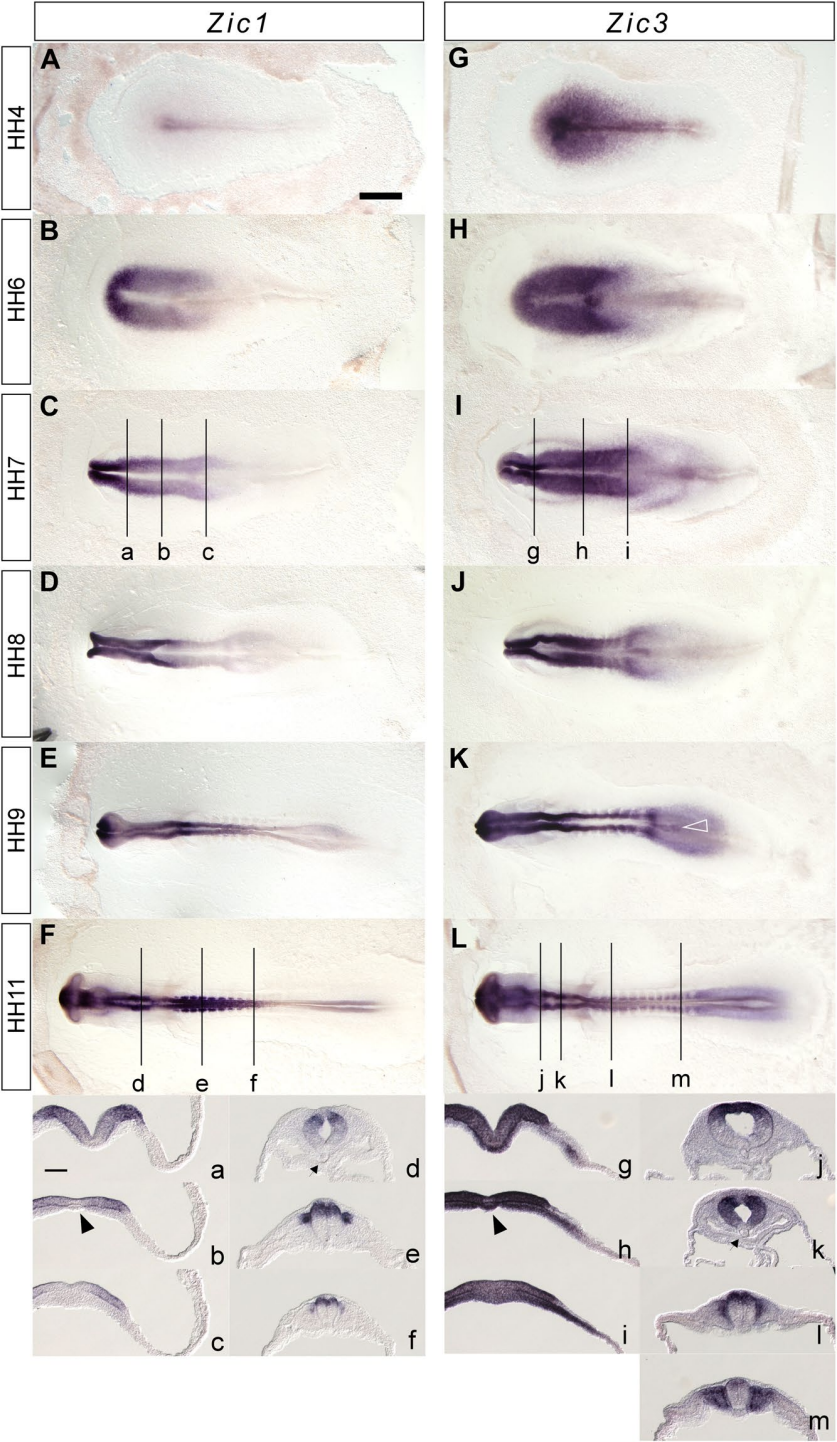
Expression of chicken *Zic1* and *Zic3* during development (**A**–**F**, a–f) *Zic1*, (**G–L**, g–m) *Zic3*. (**A,G**) HH4, (**B,H**) HH6, (**C**,**I**) HH7, (**D**,**J**) HH8, (**E**,**K**) HH9, (**F**,**L**) HH11. (a–m) Cross section images at the positions indicated in (**C**,**F**,**I** and **L**). *Arrowheads*, prechordal plates. *Arrows*, notochords. *Open arrowhead* in L, notochord progenitors. *Scale bars*, (**A**–**L**) 1 mm; (a–m) 100 μm.

We examined the expression profiles of *Zic1* and *Zic3* during early chicken development by whole-mount *in situ* hybridization (Fig. 1). At the Hamburger and Hamilton (HH)^35^ stage 4 (definitive streak stage, Fig. 1A,G), *Zic3* expression can be seen in the epiblast of the prospective anterior neuroectodermal region and along the primitive streak, but *Zic1* expression was faint at an area near the anterior primitive streak. At the head fold stage (HH6, Fig. 1B,H), both *Zic1* and *Zic3* were broadly expressed in the neural plate. *Zic1* expression was enhanced in the anterior region, whereas *Zic3* expression increased posteriorly. A dense *Zic3* signal could be seen at the node and the moderate expression continued at the primitive groove. At HH7 (Fig. 1C,I), *Zic1* and *Zic3* expression in the neural folds showed anterior-posterior profiles similar to those in the HH6 stage, but the signal was enhanced in the lateral region of the neural plate as seen in the cross sections (Fig. 1a–c,g–i). Moreover, *Zic3* expression was strong at the prechordal plate and paraxial mesoderm. At the following stage (HH8, Fig. 1D,J), *Zic3* expression was clearly observed at the somites. At later stages (HH9, Fig. 1E,K; HH11, Fig. 1F,L) of nervous systems, *Zic1* expression was accentuated at the anterior end of the telencephalic vesicle, diencephalon and hindbrain region and *Zic3* expression was enhanced in the broad region of the telencephalic vesicle, mesencephalic region, and some segments in hindbrain region. Dorsal restriction of the expression at the hindbrain and spinal cord was commonly observed for both *Zic1* and *Zic3* (Fig. 1d–f,j–m). In the mesoderm derivatives, *Zic3* expression was observed strongly at both somites and unsegmented mesoderm, and modest *Zic3* expression was observed at the notochord progenitors; whereas, *Zic1* expression was observed at dorsomedial somites. These expression profiles were consistent with previous studies which described *Zic1* and *Zic3* mRNA distribution at later stages [e.g. at HH10–24^33^, at HH7–13^34^] and distribution of the Zic proteins at HH8–23^24^.

In mesodermal development, the *Zic3* expression is predominant at these stages. The expression in unsegmented mesoderm was shown to be enhanced at presomitic mesoderm at HH9^34^. During somite development, *Zic3* is expressed in two or three of the most recently formed somites^34^. The expression initially decreased in somites followed by detectable expression in the most anterior mature somites during HH12/13 stages, whereas detectable expression was observed in the dorsomedial dermomyotome and sclerotome^34^.

### Enhancer screening in chicken embryos

**Figure 2:**
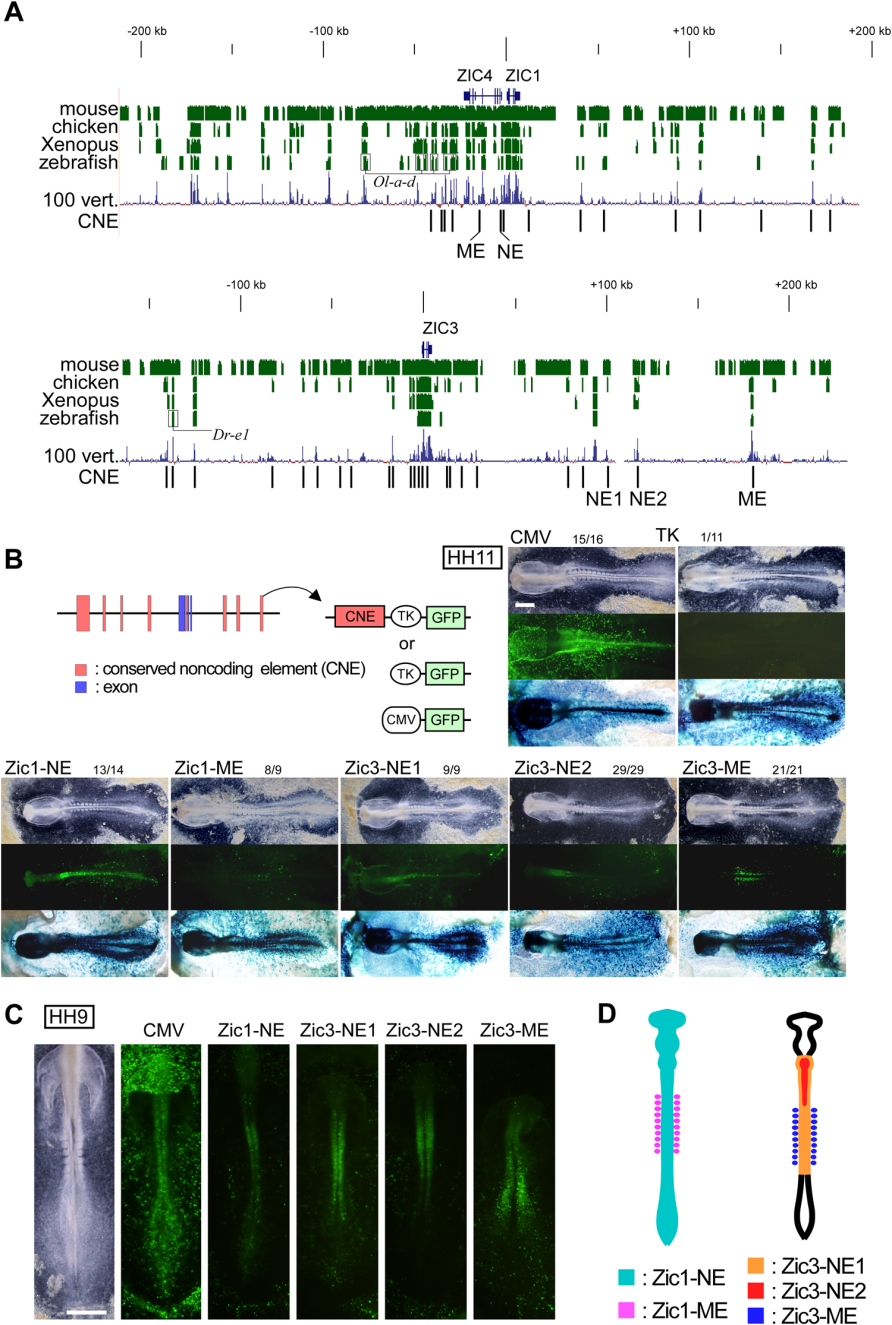
Enhancer screening in *Zic1* and *Zic3* CNEs. (**A**) Structure of human *Zic4*/*Zic1* and *Zic3*. Top indicates the scales in kilobase, centered at the transcription initiation of *Zic1* or *Zic3* the + (plus) direction is that of transcription. *Blue thin vertical lines* below gene names indicate the protein coding exons. *Clustered green vertical lines* indicate the presence of sequence similarity with the human sequence at each position of the sequences of animals indicated at the left side (mouse, chicken, Xenopus, and zebrafish). Extent of conservation is indicated as the *height of bars*. The extent of conservation among the 100 vertebrate species selected at UCSC genome browser is indicated by *100 vert*. *Black vertical lines* in the CNE line indicate the position of tested CNEs in this study. *Ol-a-d* and *Dr-e1*, known regulatory elements identified in teleost fishes^30,31^. (**B**) Structure of the reporter vector. TK, herpes simplex virus thymidine kinase promoter (minimal activity by itself); CMV, human cytomegalovirus immediate early enhancer and promoter (strong ubiquitous activity). *Pictures*, representative results of the reporter expression in chicken embryos at HH11 for *CMV*, *TK*, *Zic1-NE*, *Zic1-ME*, *Zic3-NE1*, *Zic3-NE2*, and *Zic3-ME*. (*Top*) Bright field views. (*Middle*) Reporter GFP signal at HH11 (24 h after electroporation). (*Bottom*) β-galactosidase activity staining for co-electroporated EF-LacZ to indicate the transfected cells. *Scale bar*, 1 mm. Frequencies of the reporter GFP expression/*Zic1* or *Zic3* expressing region are indicated above the pictures. Numbers indicate those of tested embryos. (**C**) Reporter GFP signals at HH9 (12 h after electroporation). Expression patterns at additional time points are shown in Supplementary Fig. S2. *Scale bar*, 1 mm. (**D**) Spatial distribution of enhancer activity in a summary.

We carried out embryo electroporation^36,37^ to identify enhancers of chicken *Zic1* and *Zic3*. The analysis focused on evolutionary conserved noncoding element (CNE) between human and chicken genome. Twenty-five and twenty-eight CNEs within 150 kb flanking regions were selected for *Zic1* and *Zic3*, respectively (Fig. 2A). The selection of distal CNEs (more than 50 kb apart) was based on the conservation between human and teleost fish sequences. The CNEs were placed upstream of the herpes simplex virus thymidine kinase (TK) promoter-driven EGFP reporter vector. TK promoter was shown to be neutral in terms of the expression in chick embryos in previous studies^36^. The CNE-TK-EGFP reporters were co-electroporated with a control vector (EF-LacZ) that directs ubiquitous expression. Electroporation was carried out at HH4, and the reporter gene expression profiles were monitored until HH11.

As a result, we observed clear enhancer activities for two CNEs for *Zic1* and three CNEs for *Zic3* (Fig. 2). These CNEs showed spatially restricted enhancer activities. In the neural tissue, Zic1-NE (neural enhancer), Zic3-NE1 (neural enhancer 1), Zic3-NE2 (neural enhancer 2) directed the reporter gene expression (Fig. 2B–D). As to the anterior-posterior (A-P) extent of expression in the HH11 neural tube (Fig. 2B,D), Zic1-NE enhancer activity was observed all along the A-P axis with enhancement in the hindbrain and the spinal cord region. Zic3-NE1 and Zic3-NE2 activities were observed in the hindbrain and the anterior spinal cord region. Along the dorsal-ventral (D-V) axis, Zic1-NE directed wide expression at the HH7 neural plate, but the expression was dorsally restricted at HH16 neural tube as determined by electroporation of the reporter constructs into neural tube at HH11 (Supplementary Fig. S1). The change in expression mimicked that of Zic proteins in the chicken neural tube development^24^. Zic3-NE1 and Zic3-NE2 directed wide expression along D-V axis in the neural tube at all the stages (Supplementary Fig. S1). In the mesodermal derivatives, Zic1-ME (mesodermal enhancer) reporter expression was observed weakly at somites, whereas Zic3-ME reporter expression was strong at both unsegmented paraxial mesoderm and somites (Fig. 2B–D, Supplementary Fig. S1), mimicking distribution of *Zic1* and *Zic3* transcripts (Fig. 1). The spatially restricted activities of all five enhancers can be traced back to 7 hours after transfection (HH6) when the enhancer activity became evident, and it continued until 24 hours post-transfection (HH11) (Supplementary Fig. S2, Fig. 2B).

Among the five CNEs, Zic1-NE homologous region in mouse genome was included in the cochlear nucleus and dorsal spinal cord enhancer region within the 2.9 kb region of *Zic1* transcription start site (Fig. 2A) that was characterized in a previous study^27^. The other CNEs were thought to be novel. Zic1-ME was located in an intron of *Zic4*, which is placed 13 kb upstream of *Zic1* in a head-to-head manner (Fig. 2A). Zic3-NE1, Zic3-NE2, and Zic3-ME were located in the 3′ flanking region of *Zic3* (Fig. 2A). In chicken, they are 45, 50, and 65 kb apart from *Zic3*, respectively. The Zic1-NE (0.9 kb), Zic1-ME (1.3 kb), Zic3-NE1 (0.4 kb), and Zic3-NE2 (0.6 kb) sequences are conserved between human and chicken but not between human and zebrafish, and Zic3-ME (1.9 kb) sequence is conserved among human, chicken, and zebrafish (Fig. 2A).

### Enhancer activities of the five CNEs in mouse embryos

To characterize the five CNEs, we tested if the homologous region in the mouse genome shows enhancer activities in mouse embryos by a transgenic reporter assay. We prepared transgenes in which the mouse CNE homologues were placed upstream of the Heat shock protein 68 (Hsp68) promoter driven-LacZ. The transgene expression profiles were examined by β-galactosidase activities in the primary transgene recipient mice at embryonic day (E) 10.5 or 11.5.

**Figure 3:**
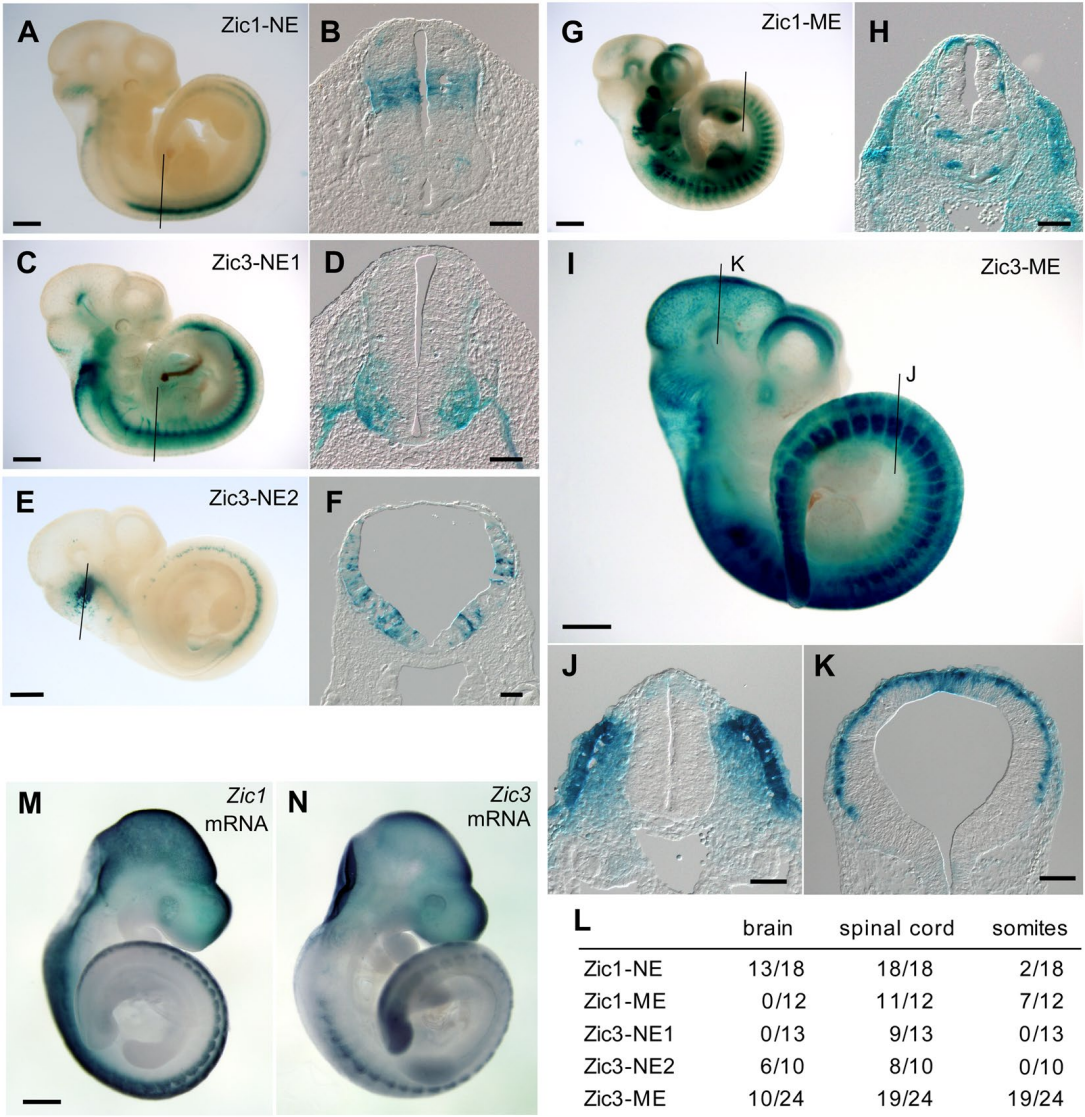
Expression of reporter gene driven by *Zic1* and *Zic3* enhancers in transgenic mice. Hsp68 promoter-LacZ was placed downstream of mouse Zic1-NE (**A,B**), Zic3-NE1 (**C,D**), Zic3-NE2 (**E,F**), Zic1-ME (**G,H**), and Zic3-ME (**I–K**). (**A,C,E,G,I**) Lateral views of E10.5 transgenic mouse embryos after X-Gal staining to detect reporter β-galactosidase expression. *Scale bar*, 1 mm. (**B,D,F,H,J,K**) Transverse sections along the *thin lines* indicated in (**A,C,E,G** and **I**), respectively. *Scale bars*, 100 μm. (**L**) Frequencies of the reporter expression in brain, spinal cord, and somites. Denominators indicate the total number of the tested embryos. (**M,N**) Whole mount *in situ* hybridization for E10.5 mice to show *Zic1* (**M**) or *Zic3* (**N**) *mRNA* distribution as references for the reporter gene assay. *Scale bar*, 1 mm.

In the mouse neural tissue, Zic1-NE gave the most robust expression in spinal cord and frequently at dorsal forebrain and midbrain (Fig. 3A,B,L). Zic3-NE2 also showed frequent expression in hindbrain and spinal cord, and Zic3-NE1 transgenes showed frequent expression in the spinal cord (Fig. 3C,D,L). All three CNEs directed the reporter gene expression in spinal cord. Although Zic1-NE transgene showed consistent activity in the dorsal spinal cord where the *Zic1* was expressed, Zic3-NE1 and Zic3-NE2 were not consistent with the endogenous *Zic3* expression^17,38^. In the mouse mesoderm derivatives, Zic3-ME frequently showed expression in the axial mesoderm including the unsegmented mesoderm, newly generated somites, and the dermomyotome of the anteriorly located mature somites (Fig. 3I,J,L), and less frequently at external layer of brain (Fig. 3I,K,L). Zic1-ME directed expression in the somite less frequently and weakly (Fig. 3G,H,L).

Thus, the expression patterns of the mouse *Zic1* and *Zic3* CNE-driven reporters were similar to those in chicken embryos, indicating that the enhancer functions are evolutionary conserved between mouse and chicken. However, the expression may not always be limited to the region of mouse *Zic1* and *Zic3* expression at the corresponding stage (Fig. 3M,N). In this regard, Zic3-ME-driven reporter appears to reliably mimic the *Zic3* expression in chicken as well as mouse^17,39^. We therefore focused on the analysis of Zic3-ME in the following experiments.

### Zic3-ME was required for the *Zic3* expression in the mesodermal tissue

**Figure 4:**
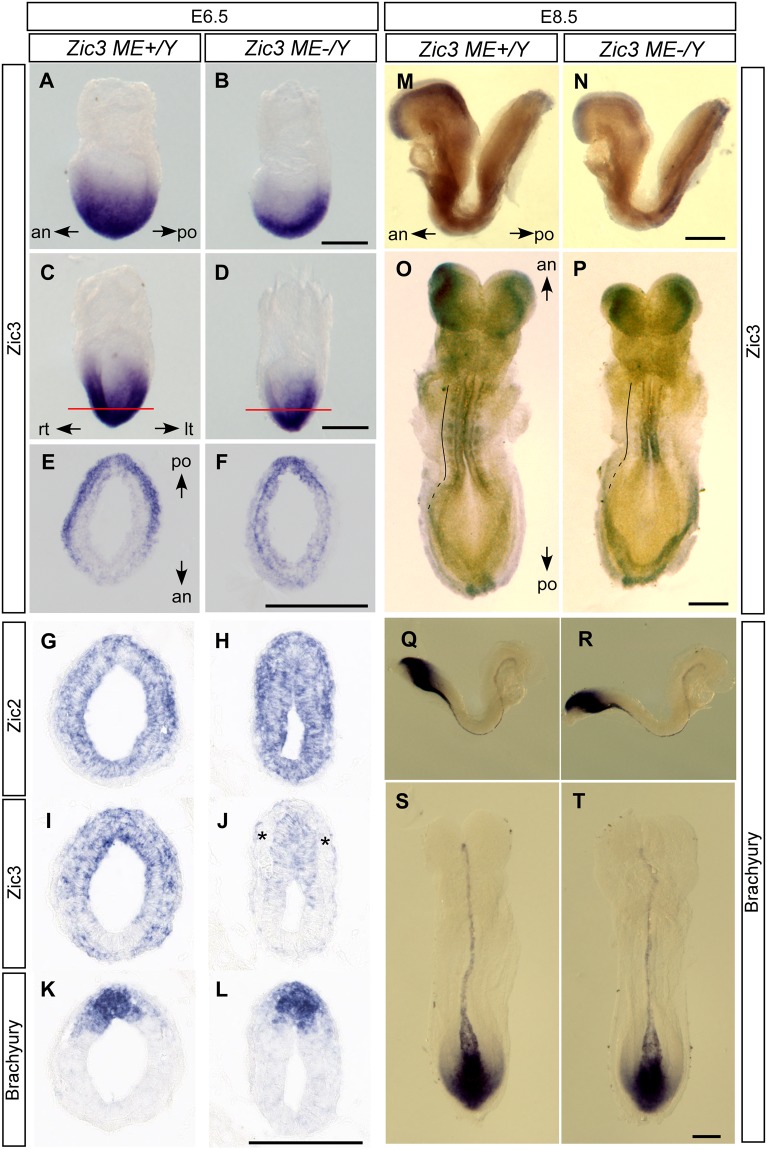
Analysis of Zic3-ME-deficient mice. Wild type (*Zic3-ME*^+/*Y*^, **A,C,E,G,I,K,M,O,Q** and **S**) and Zic3-ME-deficient mice (*Zic3-ME*^−/*Y*^, **B**,**D**,**F**,**H**,**J**,**L**,**N**,**P**,**R** and **T**) were collected at E6.5 (**A**–**L**, mid- to late-streak stage) and E8.5 (**M–T**, 9 somite-stage). *In situ* hybridization was carried out for whole-mount (**A–D** and **M–T**), and section (**E**–**L**) specimens using Zic3 (**A**–**H**,**I**,**J**,**M**–**P**), Zic2 (**G**,**H**), and Brachyury (**K**,**L**, **Q**–**T**) probes. *an*, anterior side; *po*, posterior side; *rt*, right side; *lt*, left side. (**E**,**F**) are the sections along red lines in (**C**,**D**), respectively. (**G**–**I**–**K** and **H**–**J**–**L**) are adjacent sections. *Asterisk* indicates the mesoderm where *Zic3* expression was lost. Curved lines in (**O**,**P**) indicate the location of somites (*continuous lines*) and unsegmented paraxial mesoderm (*broken lines*) at left side. *Scale bars*, 200 μm. Additional results are shown in Supplementary Fig. S4.

We next addressed the significance of *Zic3-ME* by generating *Zic3-ME* knockout (KO) mice. The mice were generated by replacing entire Zic3-ME by a loxP sequence using homologous recombination in ES cells (Supplementary Fig. S3). We examined *Zic3* expression in the E6.5 and E8.5 in Zic3-ME-deficient (*Zic3-ME*^−/*Y*^) and wild type (*Zic3-ME*^+/*Y*^) mice. By *in situ* hybridization, we found loss of *Zic3* transcript in the mesoderm of E6.5 Zic3-ME-deficient embryos (5/5), and an intact expression in the epiblast (Fig. 4B,D,F,J, Supplementary Fig. S4). At E8.5, the *Zic3* expression at somites was lost (4/4) (Fig. 4N,P, Supplementary Fig. S4). These results indicate that the Zic3-ME contains cis-regulatory elements required for the proper *Zic3* expression at mesoderm and the somites in mouse embryos at the corresponding stages^38,40^.

In the adult Zic3-ME-deficient mice, we did not observe the kinky-tail-like abnormality that is observed in Zic3-deficient mice^41,42^. *Zic2* expression in the mesoderm and the epiblast (Fig. 4H) and Brachyury expression in the primitive streak at E6.5 (Fig. 4L, Supplementary Fig. S4), the notochord, and the caudal mesoderm at E8.5 (Fig. 4R,T) remained intact in the Zic3-ME-deficient embryos. These results indicate that the altered *Zic3* expression is not due to the changes in embryonic architecture, but reflects the direct regulation *Zic3* gene expression by Zic3-ME.

### Delineation and characterization of the core region in Zic3-ME

**Figure 5:**
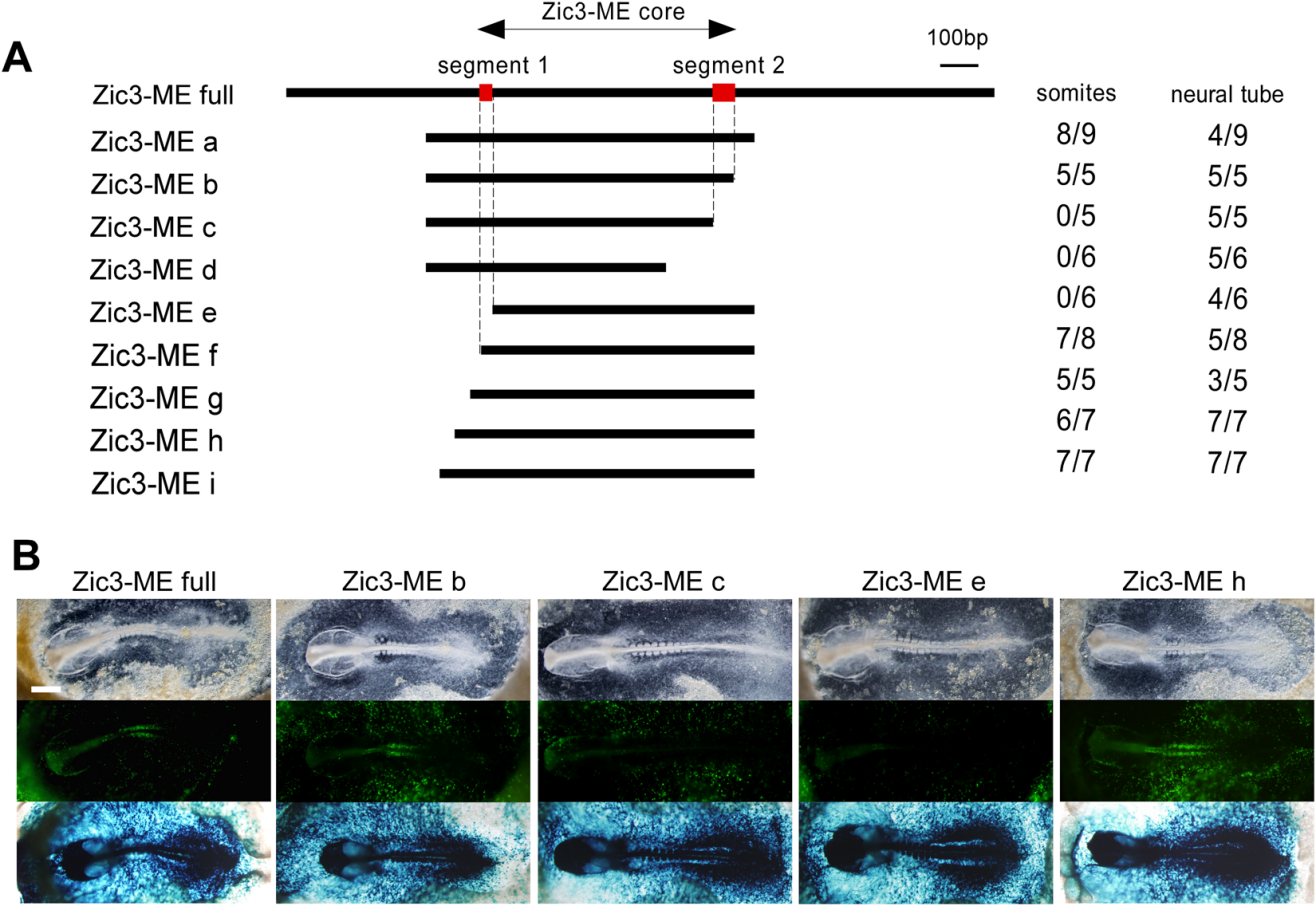
Deletion analysis of Zic3-ME in chicken electroporation assay. (**A**) Mapped Zic3-ME core region and the deletion constructs used for the assay. Right columns indicate the frequencies of the reporter expression in somite or neural tube. *Segment 1* and *segment 2* indicate the regions, of which deletion abolished the somite expression. (**B**) Representative results of the deletion assay. *Top*, bright field view; *middle*, reporter GFP signals; *bottom*, β-galactosidase activity staining to indicate the transfected cells. *Scale bar*, 1 mm.

**Figure 6:**
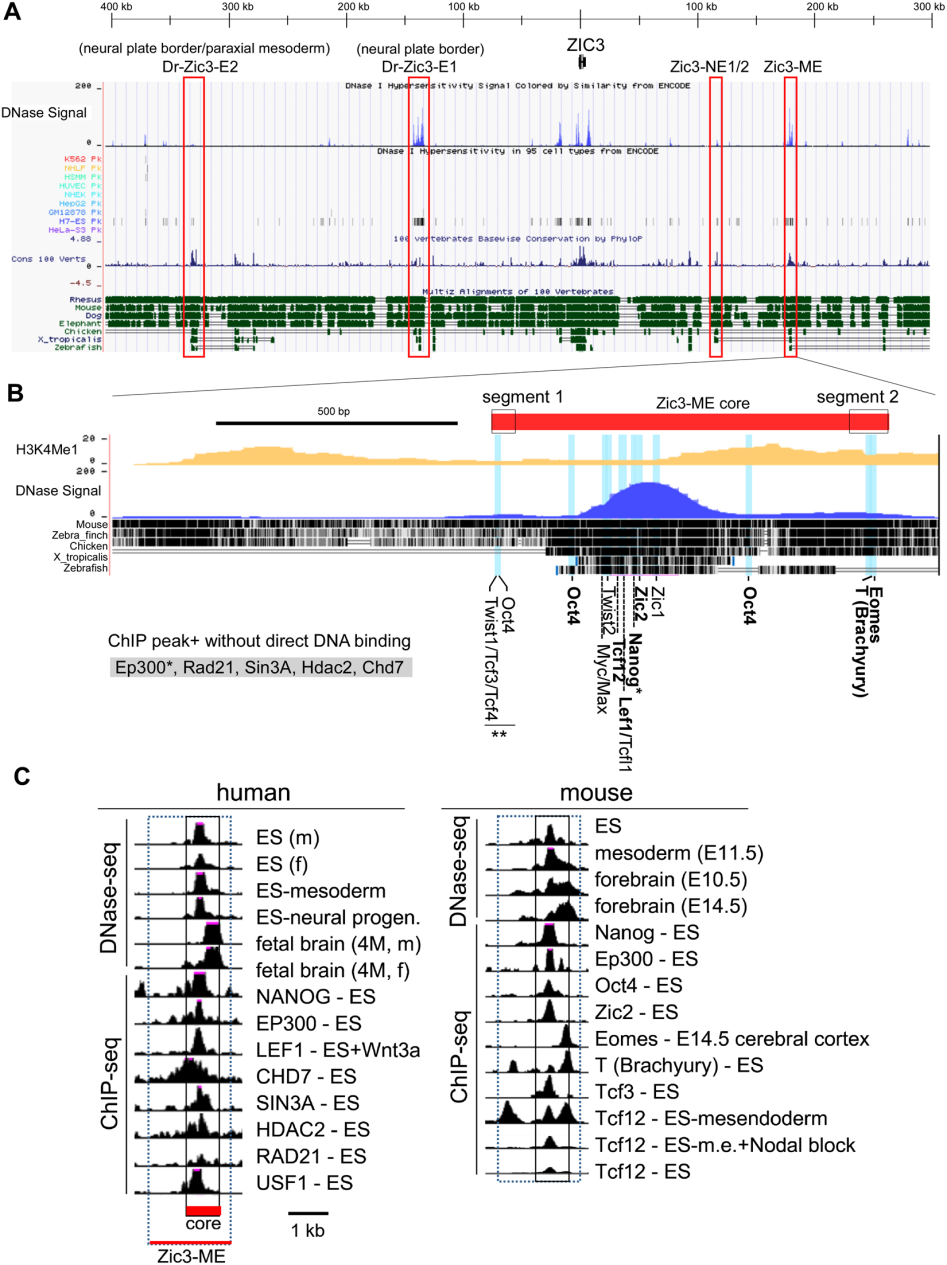
DNase HS, conservation and TF binding sites of the Zic3-ME. (**A**) *Top block*, location of DNase I HS signals. DNase I HS peaks in each cell line [top to bottom, K562 (erythroleukemia), NHLF (lung fibroblast), HSMM (myoblast), HUVEC (vein endothelial cell), NHEK (epidermal keratinocyte), HepG2 (liver cancer), GM12878 (lymphoblastoid cell), H7-ES (ES cell), and HeLa-S3 cells (cervix cancer)]. *Bottom block*, conservation in 100 vertebrate species (summary) and conservation in rhesus monkey, mouse, dog, elephant, chicken, *X. tropicalis*, and zebrafish. *Red open boxes* indicate the *Zic3* enhancers identified in this study (Zic3-NE1/2 and Zic3-ME) and in zebrafish Dr-E1 and Dr-E2^30^. (**B**) Higher magnification of Zic3-ME. *Red thick bar* indicates Zic3 ME core, From top to bottom, histone modification (H3K4Me1), DNase HS signal, and each animal species (mouse, zebra finch, chicken, *X. tropicalis*, medaka, and zebrafish). At the bottom, location of the indicated TF binding sites defined by the prediction algorithm and ChIP-seq peaks. ChIP-seq peaks of P300, Rad21, Sin3a, Hdac2, and Chd7 were identified at Zic3-ME in ES cells without predicted binding sites. *Asterisks* at TF indicate the ChIP-seq peaks identified in both human and mouse whereas *no asterisks* indicate the peaks identified in either human or mouse ChIP-seq studies. *Double asterisks* indicate the presence of the binding motifs only in the chicken sequence. *Bold letters* indicate the verification by both ChIP-seq peaks and TF binding site prediction, and *non-bold letters* indicate verification by either of the methods. (**C**) Representative DNase-seq and ChIP-seq results of human and mouse ZIC3/Zic3-ME. *Box with dotted lines* indicate the entire Zic3-ME regions and *boxes with solid lines* indicate Zic3-ME core regions. *Tcf12 (ES-mesendoderm)* and *Tcf12 (ES-m.e.(mesendoderm)* + *Nodal block)* indicate the embryoid body treated for 2 days with 100 ng/ml Activin and 100 ng/ml Activin plus 10 μM SB431542 (inhibitor of Smad2/3 phosphorylation) respectively^65^. The overall profiles including other known Zic3 enhancers and their derivations are indicated in Supplementary Fig. S6 and Table S1 respectively.

Since our findings revealed the biological significance of Zic3-ME, we defined the core region of Zic3-ME. For the deletion mutants of chicken Zic3-ME (Fig. 5), the enhancer activity was assayed using whole-embryo electroporation. Sequential deletions from both ends revealed two critical segments within a 633 bp region, which is highly conserved among vertebrates (Figs 5 and 6). We named the 633 bp region as Zic3-ME core. Zic3-ME core sequence was then queried against public DNase I hypersensitive site (DNase-HS) and chromatin-immunoprecipitation sequencing (ChIP-seq) databases.

We found that, Zic3-ME core was included in the DNase I-HS in ES cells, mesodermal cells, and developing neural cells of both human and mouse (Fig. 6A–C). In the mature tissues, brain, retina, and spinal cord cells showed Zic3-ME core in DNase-HS, but not in the heart, skeletal muscle, fat, blood, vascular cells, gut, lung, or liver (ENCODE database)^42^.

**Figure 7:**
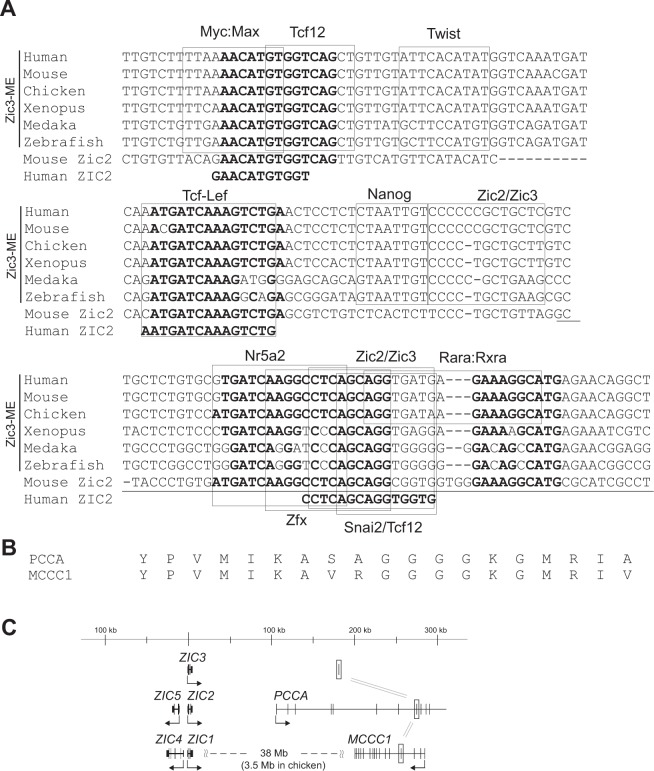
Orthologues and paralogues of Zic3-ME core sequence. (**A**) Multiple sequence alignment of most conserved region in Zic3-ME-core. From top, human, mouse, chicken, Xenopus, medaka, and zebrafish Zic3-ME nucleotide sequences are aligned. At the bottom two lines, the sequences similar to Zic3-ME in the mouse and human 3′ flanking regions of *Zic2* (Zic3-ME-like) are indicated. *Bold letters* indicate the conserved elements between Zic3-ME and its paralogue in the *Zic2* flanking region. *Underline* indicates the exon of PCCA (propionyl-CoA carboxylase alpha subunit), a heterodimeric mitochondrial enzyme. (**B**) MCCC1 (methylcrotonoyl-CoA carboxylase 1) is a paralogue of PCCA. Translated amino acid sequences of PCCA and MCCC1 are alighned. (**C**) The positions of Zic3-ME and its paralogues (*open boxes*) in the human genome. *Arrows* indicate the transcription start site and direction in each gene.

In terms of transcription factor (TF) binding, as evidenced by ChIP-seq experiments, Zic3-ME core was bound by pluripotency-associated TFs (Nanog, Oct4 [Pou5f1]), meso-endodermal differentiation associated TFs (Brachyury [T], Eomes [Tbr2], LEF1), neural differentiation-associated TFs (Zic2, Eomes), and silencer/chromatin-looping/chromatin-remodeling associated factors (Rad21, Sin3a, Hdac2, Chd7), and transcriptional cofactor (Ep300) (Fig. 6C, Supplementary Fig. S5). Accordingly, Zic3-ME contains the predicted TF binding sites for Nanog, Oct4, T, Eomes, LEF1, and Zic proteins at the sites of their ChIP-seq peaks (Figs 6 and 7, Supplementary Figs S5 and S6). In addition, we found putative TF binding sites for Twist cotrolling mesodermal differentiation^43^, Snai2 also known as slug, controlling mesoderm patterning^44^, Nr5a2 essential for primitive streak morphogenesis^45^, and Zfx controlling self-renewal of ES cells^46^; although, their ChIP-seq results are not yet available (Fig. 7).

The binding sites for Nanog, TCF-LEF, Zic2, Twist, and Myc were clustered at the most strongly conserved region where the highest DNase-HS peak lies (Figs 6 and 7). By contrast, TF binding sites for two T-box factors (T and Eomes) were located outside of the peak and included in the segment 2 (Fig. 6B,C), the deletion of which abolishes the somite expression directing activity. The segment 2 sequence is conserved in vertebrates, but not in the teleost fishes (Medaka and Zebrafish, Fig. 6). Although another region, which is necessary for the somite expression in the chicken embryos (segment 1), contained the predicted binding sites for Oct4 and Twist, the sites are not clearly conserved in mammalian species.

**Figure 8:**
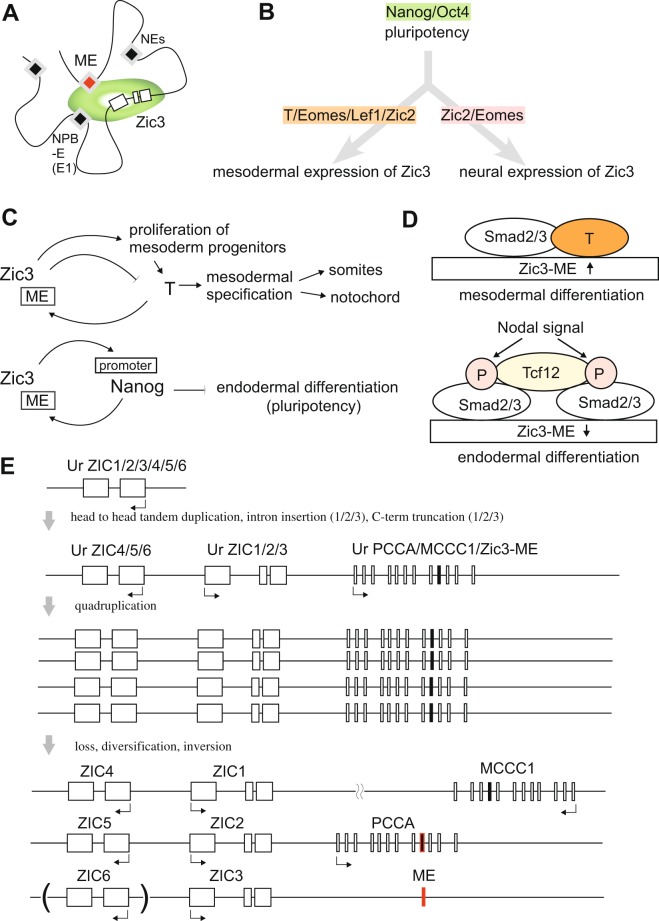
Hypotheses concerning the regulatory mechanism and evolution of Zic3-ME. (**A**) Zic3-ME and Zic3-E1 may associate with the transcriptional regulatory complex on the Zic3 coding region, based on their openness in chromatin-associating proteins, and functionalities as enhancers. This status was highly predictable in ES cells where many ChIP-seq results available, and may be involved in the cell fate specification of ES cells. (**B**) Zic3-ME was targeted by pluripotency-associated factors, mesodermal differentiation-associated factors, and neural differentiation-associated factors. They are likely to control Zic3 expression in ES, mesodermal, and neural cells in embryonic development. (**C**) Roles of Zic3-ME, based on the results of this study and the known regulatory relationships between Zic3 and T (*top*) and between Zic3 and Nanog (*bottom*). *Top*, Zic3-T feedback loop may control the expansion of immature mesodermal cells. *Bottom*, Zic3-Nanog feedback loop may be required for the maintenance of pluripotency by inhibiting endodermal inhibition. In these regulatory loops, Nanog and T associate with Zic3-ME, which in turn activates Zic3 expression. (**D**) Zic3-ME acts as a switch between mesodermal and endodermal cell fates. Activation of Nodal signaling induces mesendoderm formation, and subsequent high levels of Nodal signaling induces differentiation into definitive endoderm. Binding of Tcf12 to the T-binding site in Zic3-ME is dependent on Nodal signal and suppresses Zic3 expression. (**E**) It has been proposed that Zic paralogues appeared because of a tandem gene duplication and a subsequent quadruplication in the early vertebrate. This study predicts the presence of a common ancestral gene for PCCA, MCCC1, and Zic3-ME next to the two Zic genes as indicated in the second line. After quadruplication, PCCA, MCCC1, and Zic-ME may have diverged respectively. *Ur* indicates the common ancestor of the following genes. *Red* indicates the enhancer property, and *black* indicates the protein-coding property.

Positions of ChIP-seq peaks matched to those of the binding motifs (Fig. 6B,C). However, Ep300, Chd7, Hdac2, Rad21, and Usf1 showed their ChIP-seq peaks irrespective of the binding sequences in agreement with their known roles as cofactor or in chromatin remodeling. Tcf12 (a basic helix-loop-helix protein, also known as Heb) showed the presence of additional peaks besides the peak on the predicted binding sites. Presence of the binding-site-independent-peak depended on the Nodal signal, raising a potential controlling mechanism of Zic3-ME (see Discussion, Figs 6C and 8D, and Supplementary Figs S5 and S7). Interestingly, positions of the DNase-HS peaks with Zic3-ME are different between ES cells and fetal brain tissue both in human and mouse (Fig. 6C). Brain DNA-HS peaks are placed at the distal end of the Zic3-ME core region, overlapping the Eomes and T ChIP-seq peaks. ES DNase-HS peaks coincide with the peaks of Nanog/Oct4/Zic2/Tcf3. The difference of DNase-HS suggest that the Zic3-ME higher order chromatin structure dynamically changes during the differentiation from ES cells into immature neural cells.

### Sequence similar to Zic3-ME core exists in the 3′ flanking region of *Zic2*

In bioinformatics analysis of Zic3-ME, we noticed the presence of Zic3-ME-related sequences in vertebrate genomes. A BLAST homology search against human genome identified a sequence similar to Zic3-ME, which resides 270 kb downstream of *Zic2* (named as Zic3-ME-like, Fig. 7A,C). The sequence alignment showed that the Zic3-ME-like is similar to the most strongly conserved region of Zic3-ME core where the predicted binding sites for Myc and LEF1 are present (Fig. 7A). Surprisingly, a 59 bp region downstream of the Zic3-ME-like included the protein coding exon of propionyl-CoA carboxylase alpha subunit (PCCA) that is transcribed from the transcription start site located 100 kb downstream of *Zic2* (Fig. 7C). The human genome also contained a paralogue of PCCA, known as methylcrotonoyl-CoA carboxylase 1 (MCCC1). MCCC1 was found to be located 38 Mb downstream of *Zic1* (Fig. 7C). As the PCCA amino acid sequence encoded in Zic3-ME-like is highly conserved in MCCC1 protein (Fig. 7B), a traceable homology existed between Zic3-ME and Zic3-ME-like in the MCCC1 region. The juxtaposition of *Zic2-PCCA* is conserved widely in the vertebrate species including teleost fish (zebrafish) and cartilaginous fish (elephant shark), and the tandem array of *Zic1*-*MCCC1* was partially conserved in amniotes, including at least cow and chicken (UCSC genome browser, https://genome.ucsc.edu/). However, we did not find any traits of the enhancers (DNase-HS and histone modification) in the ENCODE database. Based on these results, we inferred the evolutionary history of Zic3-ME (See Discussion and Fig. 8E).

## Discussion

This study revealed enhancers critical for *Zic1* and *Zic3* expression in chicken and mouse embryos. The five enhancers, Zic1-NE, Zic1-ME, Zic3-NE1, Zic3-NE2, and Zic3-ME directed the spatially restricted expression in neural or mesodermal tissue. We hypothesize that they are coordinately involved in the regulation of *Zic1* and *Zic3* expression during embryonic development. However, they may be only a few pieces of enhancers necessary for the proper expression of *Zic1* and *Zic3*. This is because we targeted only the evolutionary conserved region between human and chicken genomes. In this regard, studies on *Sox2* enhancer are supportive. Full scanning of upstream and downstream 100 kb of chicken *Sox2* regions identified 26 enhancers, of which most (25 out of 26) corresponded to a fraction of conserved sequence blocks between chicken and mammalian genomes^36,47^. The exceptional one enhancer was proposed to be unique to the chicken^47^. Therefore, the conserved sequence-targeted enhancer screening as in this study would be effective to identify developmentally critical enhancers if the comprehensiveness is not absolutely needed.

Cis-regulatory elements for *Zic1* have been investigated in regions 2.9 kb upstream of mouse *Zic1* transcription start site^27^, 5.0 kb upstream of Xenopus *Zic1*, 73 kb upstream and 45 kb downstream of zebrafish *Zic1*^48^, and 106 kb upstream and 36 kb downstream of medaka fish (*Oryzias latipes*) *Zic1* gene^31^. These studies identified dorsal spinal cord and cochlear nucleus enhancer (Zic1-NE) and core promoter in mouse^27^, BMP inhibitor-responsive element in *Xenopus*^28^, and neural tube and mesodermal enhancer^31^. We showed that Zic1-NE enhancer activities are conserved in chicken and mouse. Whether or not the enhancer activities are conserved in vertebrates awaits further investigation. However, it is noteworthy that that major mesodermal enhancers in teleost fish reside distantly (11–60 kb) in the 5′ flanking region of *Zic1* and they contain the four blocks of sequences conserved among teleost fishes and mammals^31^ (Ol-a-d in Fig. 2A). Insertion of a large transposon between the teleost mesodermal enhancers and *Zic1* results in mesoderm-specific loss of *Zic1* expression^31^. If the Ol-a-d enhancer activities are conserved in chicken or in mouse, Zic1-ME may control the mesodermal expression cooperatively with them.

Two cis-regulatory elements for have been reported in the distant region upstream of zebrafish *Zic3*^30^. These enhancers are distinct from Zic3-NE1, Zic3-NE2, and Zic3-ME analyzed in this study. Zic3-E1 (16 kb upstream of zebrafish *Zic3*, Dr-e1 in Fig. 2A) directs reporter gene expression at the neural plate border, and E2 (53 kb upstream) enhancer activity is present in the neural plate border, anterior neural plate, and posterior paraxial mesoderm at an early stage (12 hours post fertilization), anterior dorsal neural tube, and posterior paraxial mesoderm at a later stage (24 hours post fertilization). E1, but not E2, shows clear enhancer signatures in terms of an epigenetic modification peak, summed TF-binding peak, and DNase-HS peaks in human or mouse genomes (Fig. 6 and Supplementary Fig. S5). Zic3-ME and Zic3-E1 may associate with the transcriptional regulatory complex on the *Zic3* coding region in mammalian embryos (Fig. 8A), because humans and/or mouse Zic3-E1, *Zic3* coding region, and Zic3-ME are all in the open chromatin structure as evidenced by DNase-HS peaks (Supplementary Fig. S5) and are all associated with the active and primed enhancer signature (H3K4Me1)^49^ (ENCODE database). Furthermore, all three regions are bound by proteins associated with chromatin remodeling factors (Chd7 and Hdac2) or the factor defining higher order chromatin architecture (Rad21)^50,51^ in ES cells (Fig. 6 and Supplementary Fig. S5).

In the differentiation processes of mesoderm and its derivatives, Zic3-ME binding of TFs related to the mesodermal differentiation may play important roles (Fig. 8B). These include the LEF-TCF TFs that mediate Wnt-β-catenin signaling, which have well known roles in mesodermal differentiation^52–54^. Brachyury (T), a direct target of Wnt-β-catenin signaling^55^, is a T-box type mesodermal TF that directly activates the mesodermal *Zic3* expression in *Xenopus* embryos^26,56^. Another T-box factor, Eomes, is essential for mesoderm formation and the recruitment of prospective mesodermal cells to the primitive streak^57^. Zic2, another member of the Zic family, has been shown to increase the paraxial mesoderm progenitors at the primitive streak cooperatively with Zic3^38^. In addition, Zic2 and Zic3 are also required for precise somitogenesis^38^. These results indicate that Zic3-ME acts as a highly integrated hub to control mesodermal development.

In previous studies, Zic3 overexpression suppresses Xenopus Brachyury expression and Wnt-β-catenin signaling in mesoderm, resulting in an impaired notochord development^58^, whereas Brachyury upregulates Zic3 mesodermal expression^26^. On the other hand, both mouse Zic2 and Zic3 are required to increase the paraxial mesodermal cells in mice^38^. The results are consistent if we assume that the role of Zic3 is temporally limited to enhancing mesoderm generation, and not in the generation of the notochord at a later stage. This idea is also supported by the expression profile of the chicken Zic3 that is expressed in the notochord progenitor but not in the notochord itself. Collectively, the Zic3-Brachyury (T) regulatory loop (Fig. 8C) may be critically involved in controlling mesodermal development.

Zic3-ME also contains the brain enhancer activity (Fig. 3I,K). The expression of *Zic3* in the brain was detected at E10.5 (Fig. 3N, and ref.^17^), and Eomes (Tbr2) is expressed in the brain beginning around E10.0^59^. Together with the shift of a ChIP-seq peak to the Eomes binding site during neural differentiation (Fig. 6C), we speculate that Eomes could be involved in the brain enhancer activity of Zic3-ME (Fig. 8B).

Although this was not directly assayed in this study, Zic3-ME may be involved in controlling pluripotent stem cells. Because many DNase-HS studies indicate that Zic3-ME is in the open chromatin structure in human and mouse ES/iPS cells (Fig. 6, Supplementary Fig. S5, ENCODE database), and Zic3-ME is bound by pluripotency-associated TFs (Nanog and Oct4) (Fig. 6, Supplementary Fig. S5). In ES cells, knockdown of Oct4, but not of Nanog, suppresses *Zic3* expression^60,61^. Oct4 interacts with Nanog and associates with multiple transcriptional repression complexes including Sin3A complex^62^. Zic3-ME is likely to be targeted by the Sin3A/deacetylase complex, which has been shown to cooperate functionally with Nanog to promote pluripotency^63^. Therefore, we speculated that Nanog was involved in fine-tuning Zic3-ME function.

On the other hand, Zic3 protein binds to a promoter region of Nanog and directly upregulate Nanog expression in ES cells^64^ (Fig. 8C). Moreover, Zic3 and Nanog prevent endodermal lineage specification, and *Zic3* expression was required for ES cell pluripotency^6^. Thus, the Zic3 and Nanog feedback loop as a whole would be associated with the establishment of pluripotency (Fig. 8C). Further investigation on the regulation of Zic3-ME in ES cells would be beneficial for better understanding of the mechanism underlying pluripotency.

Additionally, the ChIP-seq results clarified the role of Zic3-ME regulation in the mesoderm-definitive endoderm bifurcation (Fig. 8D). Tcf12 (also called Heb, a basic helix-loop-helix [bHLH] E protein that forms a heterodimer with another bHLH TF) binds Zic3-ME in a partially Nodal signaling-dependent manner (Fig. 6C, Supplementary Fig. S5)^65^. Depletion of Tcf12 in mesendodermal cell results in mesodermal differentiation^65^. Tcf12 has been proposed to be a Smad2/3 cofactor^66^ that link Nodal signaling^65^. T also interacts and collaborates with Smad2/3, but mediates mesoderm formation^67^. Tcf12 and T could alternatively associate with Zic3-ME, since we found ChIP-seq peaks for Smad2/3, Tcf12, and T at the same site in Zic3-ME (Fig. 6C, Supplementary Fig. S5). Furthermore, Tcf12 knockdown increases Zic3 expression in mesendodermal cells (Supplementary Fig. S7). Combining the fact that *Zic3* knockdown in ES cells results in the endodermal differentiation^6^, Zic3-ME may be involved in the switching between mesodermal and endodermal differentiation of ES cells (Fig. 8D).

Finally, based on the presence of additional Zic3-ME-related sequences, we hypothesize the origin of Zic3-ME as follows. Ancestral Zic3-ME may have existed in the vertebrate ancestor after the tandem head-to-head duplication^5^ (Fig. 8E). At this point, prototypal ME may have coexisted with a protein coding exon of common ancestor gene for PCCA/MCCC1 in the 3′ flanking region of *Zic1/2/3* common ancestor. The whole genome quadruplication and subsequent loss of one copy^5^ may have generated two additional sets of the “head-to-head tandem *Zic* genes”, PCCA/MCCC1/ME. Thereafter, diversification of PCCA/MCCC1/ME may have occurred where Zic3-ME kept and presumably acquired additional TF binding sites sequences, *PCCA* exon retained the protein coding sequence with remnant TF binding sequences, and *MCCC1* retained the protein coding information, but lost all of the TF binding sequences. We think Zic3-ME may provide us an intriguing model of enhancer evolution, awaiting further validation by experimental and computational molecular phylogenetic analyses.

## Methods

### Animals

All animal experiments were approved by Animal Experiment Committees at the RIKEN Brain Science Institute and Animal Care and Use Committee of Nagasaki University, and carried out in accordance with the guidelines for animal experimentation in RIKEN and Nagasaki University.

### Bacterial Artificial Chromosome (BAC) clones and sequencing

Original chicken *Zic* cDNA fragments were obtained by low stringency screening of chicken embryo cDNA library^24^. *Zic* BAC clones- CH261-95N3 (*Zic1*), CH261-11A12 (*Zic2*), CH-98E11 (*Zic3*) were isolated by high-stringency hybridization using high-density colony hybridization filters; the BAC filter and clones were purchased from BACPAC Resources (Children’s Hospital Oakland Research Institute, Oakland, CA, USA). The hybridization, sequencing, and sequence analysis were performed as described previously^5^.

### Plasmid construction

We used pd2EGFP-1 in which a herpes simplex virus thymidine kinase promoter from pRL-TK (Promega) was inserted at its 5′ multiple cloning site. CNEs were PCR cloned from chicken BAC clones and inserted upstream of the TK-d2EGFP unit. Control EF-LacZ vector was generated by inserting LacZ cassette from pMC1871 (Amersham) and oligonucleotide containing an initiation methionine into pEF-BOS vector^68^. For the transgenic assay in mouse, CNEs were inserted into an Hsp promoter-driven β-galactosidase reporter cassette^69^.

### *In situ* hybridization

*In situ* hybridization (ISH) was performed essentially as described^38,58^.

### Chicken embryo electroporation and enhancer assay

For the description of the chicken developmental stages, Hamburger and Hamilton stages were used^35^. Chicken embryo electroporation and whole embryo culture was carried out essentially as described^36,37^. Fertilized chicken eggs were purchased from Inoue Egg Farm (Kanagawa, Japan). Briefly, fertilized eggs were incubated at 38 °C for 26 hours. The chicken embryos at HH4 were excised, and attached to a sterilized paper filter with hole. The yolks attached on embryos were rinsed with Hank’s balanced salt. The embryos were placed upside down on a 2 × 2 mm platinum plate electrode (cathode) on a dish (CUY700P, Nepagene). After one microliter of transfection cocktail (2 μg/μl CNE-TK-d2EGFP, 1 μg/μl of pEF-LacZ, 0.5 μg/μl fast green was injected into the space between the blastoderm and vitelline membrane using a glass pipette, electric pulses (10 V, 50 ms, 100 ms intervals, five times) were delivered using the electroporator (CUY21, Nepagene). The transfected embryos were placed on the agarose-albumin plate^36^, covered with yolk supernatant diluted in Hank’s solution and incubated at 38 °C.

### Generation and analysis of transgenic and Zic3-ME KO mice

Transgenic mice were generated at Nihon SLC (Shizuoka, Japan). A BAC clone containing *Zic3* was purchased from the BACPAC Resources of the Children’s Hospital, Oakland Research Institute. Zic3-ME targeting vector was constructed to replace Zic3-ME with a neomycin resistance gene cassette flanked by a loxP sequence (Neo). Homologous genomic DNA with the Neo cassette was joined with a diphtheria toxin A cassette for negative selection. Linearized targeting vectors were electroporated into C57BL/6J ES cells (EmbryoMax, Millipore) and homologous recombinants were isolated by G418 selection. The ES clones were screened by Southern blot analysis (Supplementary Fig. S3). Correctly targeted ES clones were injected into blastocysts of C57BL/6J mice, which were then used to produce chimeric mice. After confirmation of germ line transmission, the Neo cassette was removed by crossing mice that had germ line transmission with transgenic mice expressing Cre recombinase in germ cells^70^. The Cre recombination was confirmed by PCR and Southern blot analyses. Mutant animals were genotyped by PCR using DNA and the following primers: Forward primer F1 (5′-CTATGCTCATCGCTTTCGCCATCTAA-3′) and Reverse primer R1 (5′-ATTTTCACGGCCAGCAGTGTTGATAG-3′) for the knockout (KO) allele; and Forward primer F2 (5′-TCTGTGAGGGGATGTTGGAT-3′), and Reverse primer R2 (5′-CCCTGCAGCATGGAGATAAG-3′) for the wild type (WT) allele.

### Bioinformatics analysis

ChIP-seq peaks were displayed using the Cistrome database (http://cistrome.org/db/#/)^71^, ENCODE database (https://www.encodeproject.org/)^72^, and GTRD (http://gtrd.biouml.org)^73^. A computer-assisted Zic family binding sites search was carried out at JASPAR database (http://jaspar.genereg.net/) and using the MatInspector program (Genomatix, Munich, Germany). A homology search was carried out with the NCBI BLAST (https://blast.ncbi.nlm.nih.gov/Blast.cgi). General analysis was done using UCSC genome browser (https://genome.ucsc.edu/) and Ensembl genome browser (https://www.ensembl.org/).

## Electronic supplementary material

Supplementary information

## Data Availability

The sequences newly defined in this study were deposited at the DDBJ/GenBank/EMBL database under the following accession numbers: LC377844 (CH261-98E11), LC377845 (CH261-95N3), LC377846 (CH261-11A12), LC377847 (Zic3-ME), LC377848 (Zic3-NE1), LC377849 (Zic3-NE2), LC377850 (Zic1-NE), and LC377851 (Zic1-ME).
