# Supplementary material for: An Evolutionarily Conserved Mesodermal Enhancer in Vertebrate Zic3
Source: Sci Rep. 2018 Oct 8;8:14954. doi: 10.1038/s41598-018-33235-y (PMC6175831; doi:10.1038/s41598-018-33235-y)
Supplement: Supplementary file 1 — Supplementary information [file 41598_2018_33235_MOESM1_ESM.pdf]

Supplementary information for Yuri S. Odaka, Takahide Tohmonda, Atsushi Toyoda, and Jun Aruga
"An evolutionarily conserved mesodermal enhancer in vertebrate Zic3"

Supplementary Table S1
Data resources for Fig. 6C and Supplementary Fig. S5

DATABASE	species	cell type (Fig. 6C)	Exp. Type	Biosamples	Detailed information, database accession number
Fig. 6C and Suppl. Fig. S5 top panel					
ENCODE	human	ES (m)	DNaseI HS	ES-H1-hESC	DNase-seq of Homo sapiens male embryo H1-hESC stem cell - ENCSR000EMU - GSM736582
ENCODE	human	ES (f)	DNaseI HS	ES-H9	DNase-seq of Homo sapiens female embryo (5 days) H9 stem cell - ENCSR275ICP
ENCODE	human	ES-mesoderm	DNaseI HS	ES(H7-hESC)-derived mesodermal progenitors	DNase-seq of Homo sapiens female embryo cardiac mesoderm in vitro differentiated cells - ENCSR000EMW - GSM1024755
ENCODE	human	ES-neural progen.	DNaseI HS	ES(H9)-derived neural progenitors	DNase-seq of Homo sapiens female embryo (5 days) neural progenitor in vitro differentiated cells originated from H9 - ENCSR963ALV
ENCODE	human	fetal brain (4M,m)	DNaseI HS	brain	DNase-seq of Homo sapiens male embryo (122 days) brain tissue - ENCSR649KBB
ENCODE	human	fetal brain (4M, f)	DNaseI HS	brain	DNase-seq of Homo sapiens female embryo (117 days) brain tissue - ENCSR309FOO
CISTROME	human	NANOG	ChIP-seq	ES-H1-hESC	NANOG ChIP-seq of Homo sapiens male embryo H1-hESC stem cell -
CISTROME	human	EP300	ChIP-seq	ES-H1-hESC	EP300 ChIP-seq of Homo sapiens male embryo H1-hESC stem cell - ENCSR000BKK
CISTROME	human	LEF1	ChIP-seq	ES-H1-hESC	LEF1 ChIP-seq of Homo sapiens male embryo H1-hESC stem cell - GSM1579344 - PMID: 25936800
CISTROME	human	LEF1	ChIP-seq	ES-H1-hESC treated with Wnt3a (200 mg/ml, 4 h)	LEF1 ChIP-seq of Homo sapiens male embryo H1-hESC stem cell - GSM1579344 - PMID: 25936800
CISTROME	human	CHD7	ChIP-seq	ES-H1-hESC	CHD7 ChIP-seq of Homo sapiens male embryo H1-hESC stem cell - ENCSR000AVA
CISTROME	human	SIN3A	ChIP-seq	ES-H1-hESC	SIN3A ChIP-seq of Homo sapiens male embryo H1-hESC stem cell - ENCSR000BIS
CISTROME	human	HDAC2	ChIP-seq	ES-H1-hESC	HDAC2 ChIP-seq of Homo sapiens male embryo H1-hESC stem cell - ENCSR000AVB
CISTROME	human	RAD21	ChIP-seq	ES-H1-hESC	RAD21 ChIP-seq of Homo sapiens male embryo H1-hESC stem cell - ENCSR000ECE
CISTROME	human	USF1	ChIP-seq	ES-H1-hESC	USF1 ChIP-seq of Homo sapiens male embryo H1-hESC stem cell - ENCSR000BIU
Suppl. Fig. S5 middle panel					
GTRD	human	NANOG	ChIP-seq	ES-H1-hESC, ES-HUES64	meta-clusters of peaks called by MACS;GEM;PICS;SISSRS
GTRD	human	TCF12	ChIP-seq	LoVo	meta-clusters of peaks called by MACS;GEM;PICS;SISSRS
GTRD	human	SMAD2	ChIP-seq	ES-H9, K562, LoVo	meta-clusters of peaks called by MACS;GEM;PICS;SISSRS
GTRD	human	SMAD3	ChIP-seq	A549, ES-BGO3, ES-H9, NCI-H441, LX-2	meta-clusters of peaks called by MACS;GEM;PICS;SISSRS
GTRD	human	T	ChIP-seq	ES(H9)-derived endodermal progenitors<br>ES(H9)-derived mesodermal progenitors	meta-clusters of peaks called by MACS;GEM;PICS;SISSRS
GTRD	human	EOMES	ChIP-seq	ES-H9, ES-HUES64, LoVo	meta-clusters of peaks called by MACS;GEM;PICS;SISSRS
Fig. 6C right and Suppl. Fig. S5 bottom panel					
ENCODE	mouse	ES	DNaseI HS	ES-E14	DNase-seq of Mus musculus strain 129/Ola male embryo ES-E14 stem cell - ENCSR000CMW
ENCODE	mouse	mesoderm (E11.5)	DNaseI HS	axial somatic and lateral plate mesoderm from eviscerated headless, limbless embryos	DNase-seq of Mus musculus CD-1 male embryo (11.5 days) mesoderm tissue - ENCSR000CNO
ENCODE	mouse	forebrain (E10.5)	DNaseI HS	forebrain	DNase-seq of Mus musculus strain B6NCrl mixed sex embryo (10.50 days) forebrain tissue - ENCSR756SPS
ENCODE	mouse	forebrain (E14.5)	DNaseI HS	forebrain	DNase-seq of Mus musculus strain B6NCrl mixed sex embryo (14.5 days) forebrain tissue - ENCSR337EDG
ENCODE	mouse	Nanog	ChIP-seq	ES-E14TG2a.4	NANOG ChIP-seq of Mus musculus strain 129/Ola male embryo (3.5 days) E14TG2a.4 stem cell - ENCSR779CZG
ENCODE	mouse	Ep300	ChIP-seq	ES-Bruce4	EP300 ChIP-seq of Mus musculus strain Bruce4 male embryo ES-Bruce4 stem cell - ENCSR000CCD
CISTROME	mouse	Pou5f1	ChIP-seq	ES-129/Sv-derived	WT_ESC_OCT4_CHIPSEQ_REP2 GSM1910646 - PMID: 28212747
CISTROME	mouse	Zic2	ChIP-seq	ES-V6.5	ZIC2_REP2 GSM1499117 - PMID: 25699711
CISTROME	mouse	Eomes	ChIP-seq	E14.5 Cerebral cortex C57BL/6	TBR2 CHIP GSM1553879 - PMID: 27600842
CISTROME	mouse	T (Brachyury)	ChIP-seq	ES-D3	BRACHYURY CHIPSEQ ANTIBODY #1 GSM1327412 - PMID: 24616493
CISTROME	mouse	Tcf3	ChIP-seq	ES-E14TG2a	TCF3 CHIP-SEQ DAY 0 ES GSM1782923 - PMID: 27889317
CISTROME	mouse	Tcf12 (mesoendoderm)	ChIP-seq	ES-CGR8-derived embryoid body was treated with Activn A (100 ng/ml, 48 h) in chemically defined media	ENDODERM_HEB_CHIPSEQ GSM1288316 - PMID: 25775035
CISTROME	mouse	Tcf12 (m.e.+Nodal block)	ChIP-seq	ES-CGR8-derived embryoid body was treated with Activn A (100 ng/ml, 48 h) and SB431542 (TGF-beta signaling inhibitor, 10 μM, 24 h) in chemically defined media.	ENDODERM+SB_HEB_CHIPSEQ GSM1288318 - PMID: 25775035
CISTROME	mouse	Tcf12 (ES)	ChIP-seq	ES-CGR8 treated with SB431542 (10 μM, 24 h).	ESC+SB_HEB_CHIPSEQ GSM1288313 - PMID: 25775035

Embryonic stem cells are preceded by "ES-".
Non-embryonic stem cells are A549 (lung carcinoma), K562 (myelogenous adenocarcinoma), LoVo (colorectal adenocarcinoma), LX-2 (hepatic stellate cells), and NCI-H441 (squamous cell lung carcinoma).

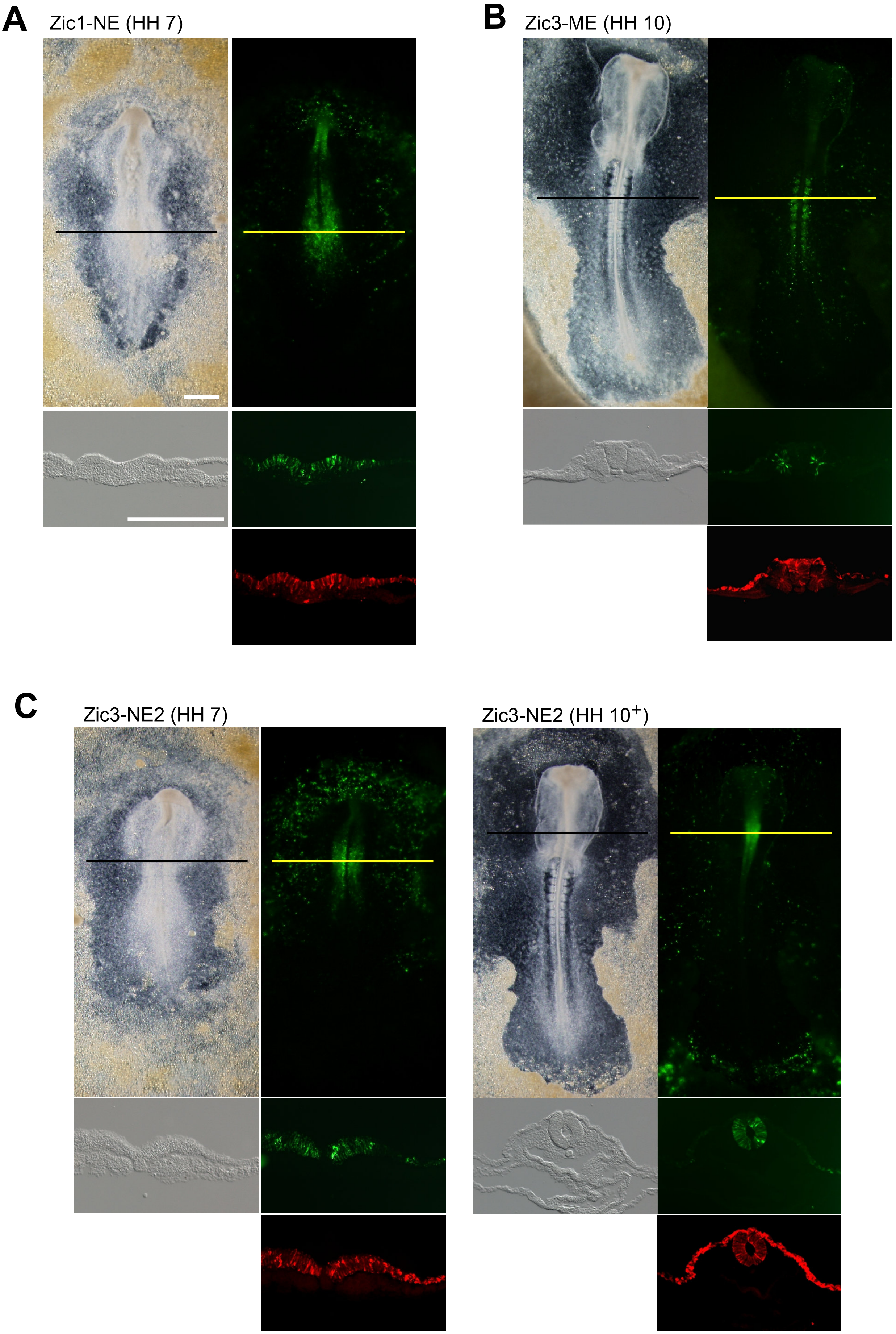

Supplementary Figure S1-1
Reporter GFP signals in cross sections.
(A) Zic1-NE, (B) Zic3-ME, and (C) Zic3-NE reporter constructs were electroporated as in Fig. 2. In each panel set, *left column* indicate bright field top view of whole embryo (*top*) and cross section DIC image, and *right column* indicate the reporter derived GFP signals (*top*, top view; *middle*, cross section) and co-electroporated CMV-DsRed derived signals (*bottom*). The cross sections are prepared by sectining through the *horizontal lines* in the top panels. *Scale bar*, 1 mm.

D

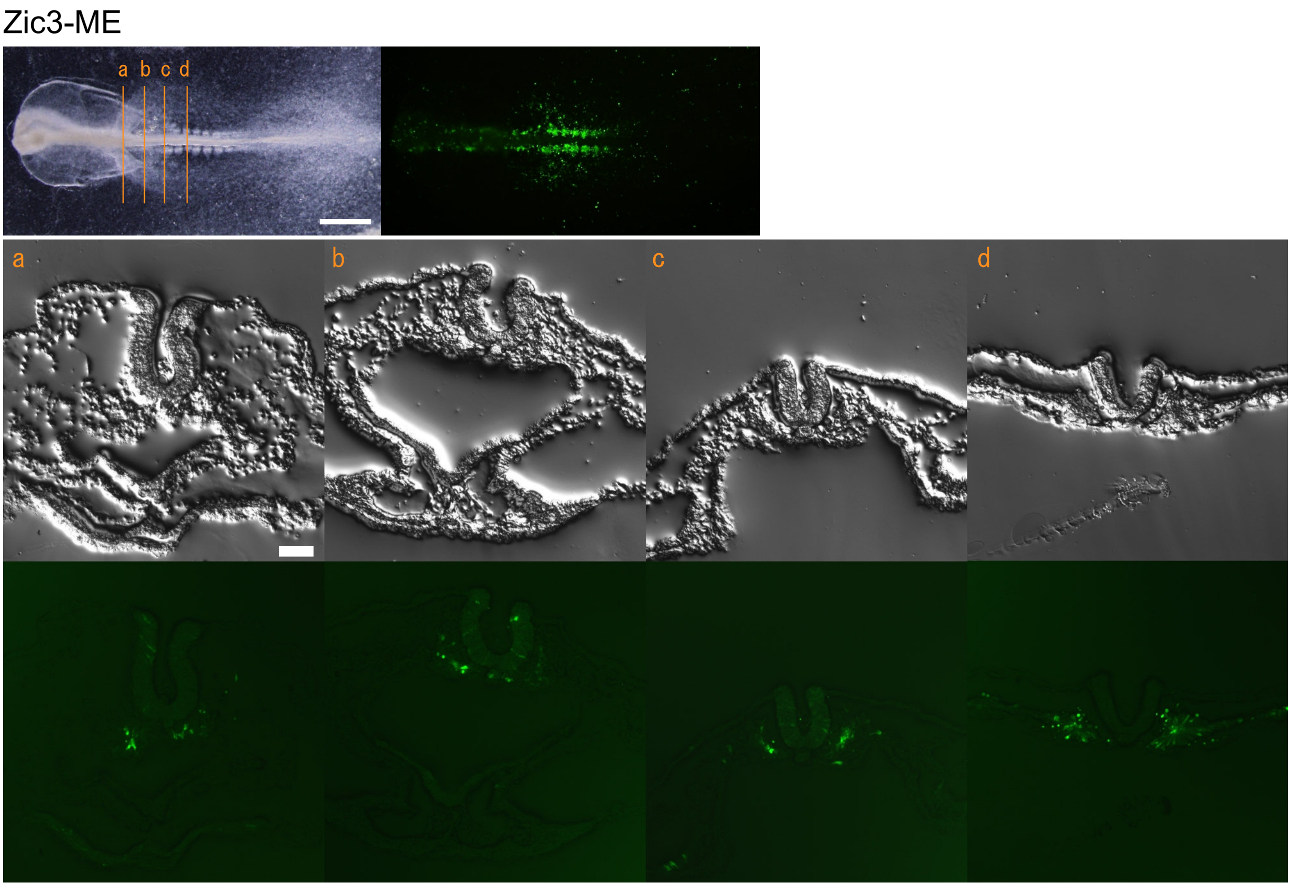

E

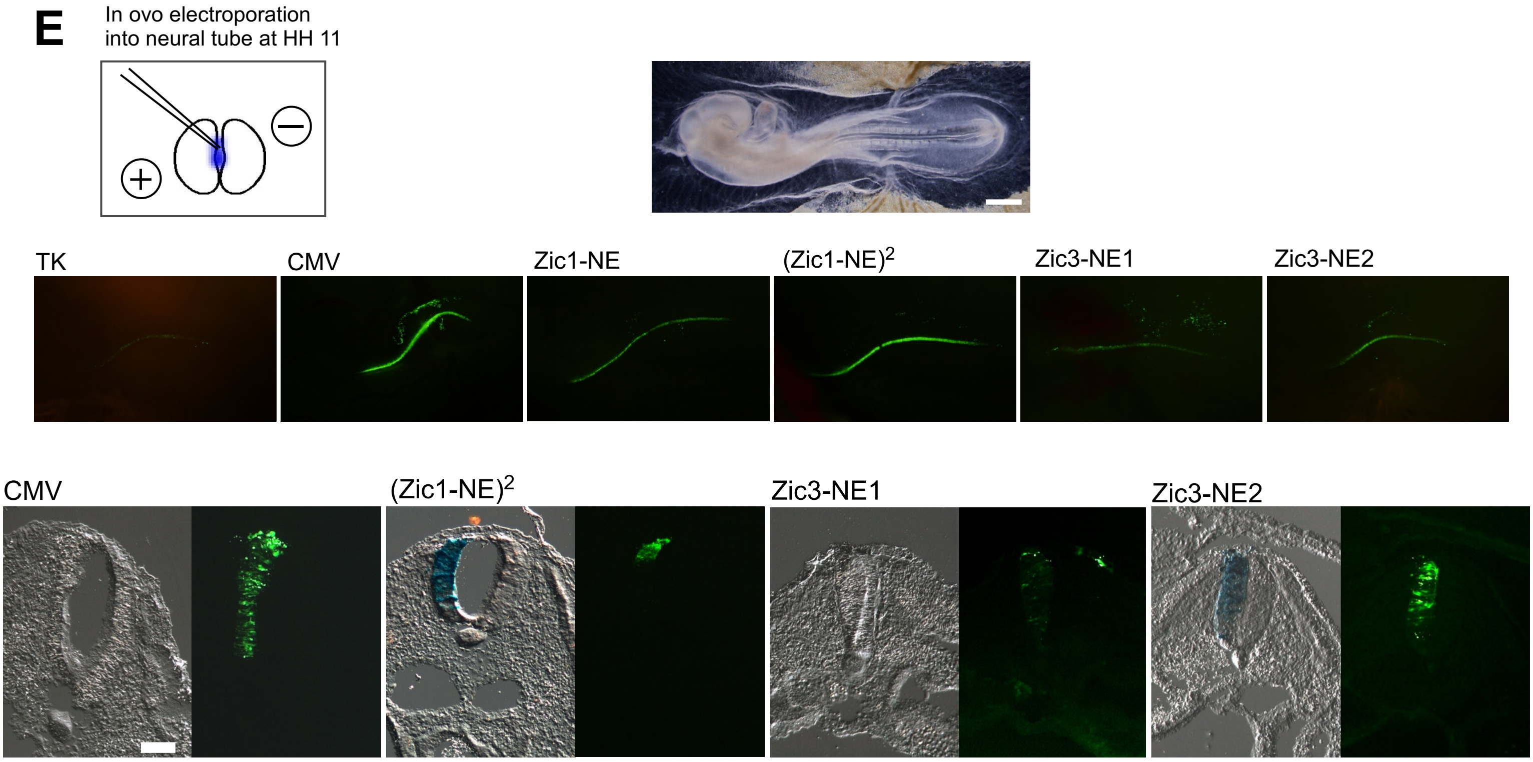

Supplementary Figure S1-2
Reporter GFP signals in cross sections.
(D) Zic3-ME reporter construct was electroporated as in Fig. 2. The embryo was sectioned through at four rostrocaudal levels (*a*-*d*). The DIC image and the GFP signals and indicated. (E) The CNE-reporter constructs were electroporated into the hemilateral neural tube after injecting the plasmid solutioin into the closing neural tube at HH11 as in *top illustration*. The embryos were fixed after 18 h (HH 16, *top picture*). *Middle line pictures* indicate the GFP signals in the top view of whole embryos. In the *bottom line pictures*, four pairs of bright field DIC images (*left*) and GFP signals (*right*) are placed where top labels indicate the name of reporter constructs. *(Zic1-NE)*^{2} indicates tandemly duplicated Zic1-NE. *Blue signals* in the bright field images derived from co-electroporated EF-LacZ. The cross sections are prepared by sectining through the horizontal lines in the top panels. *Thin scale bars*, 1 mm; *thick scale bars*, 100 µm.

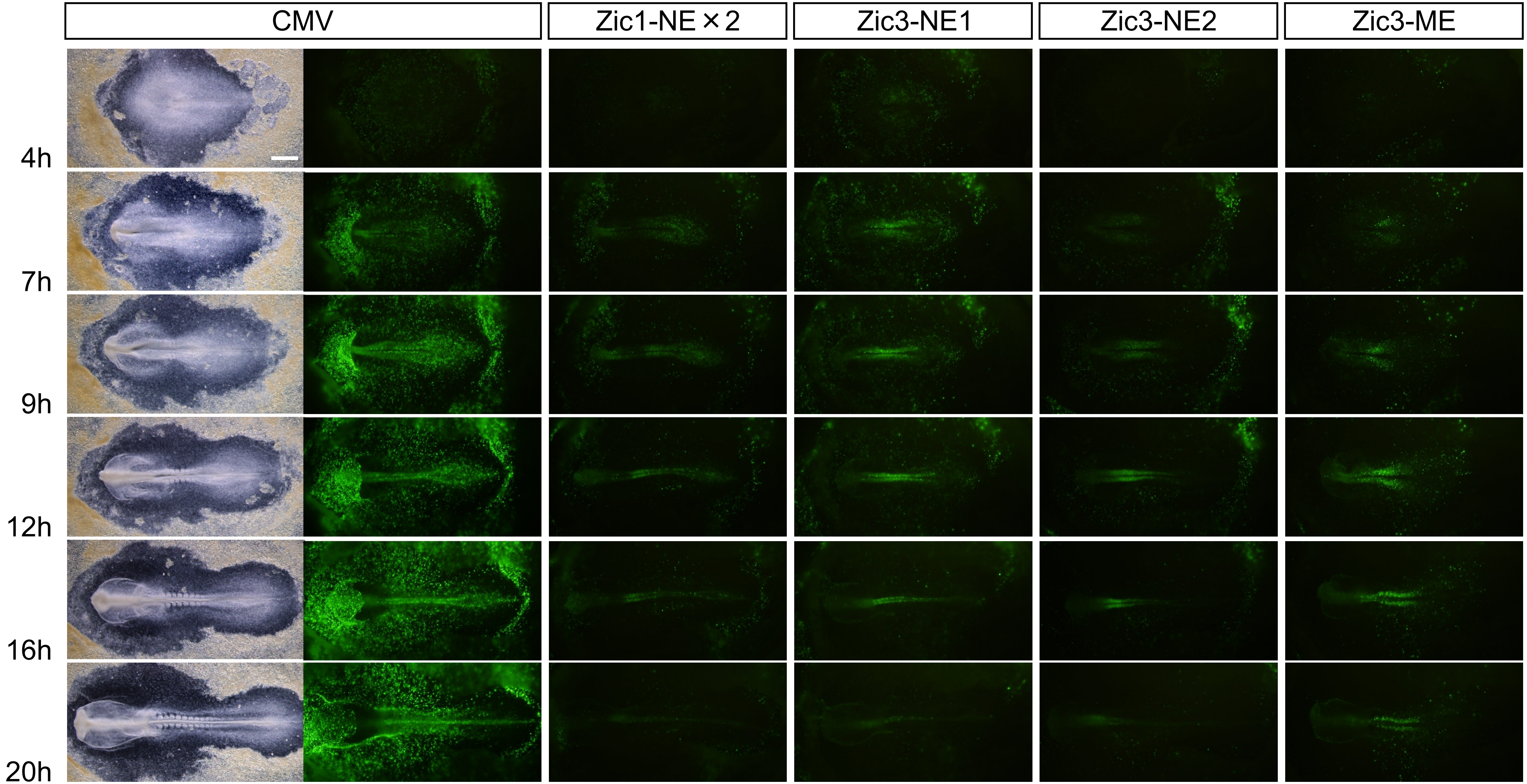

Supplementary Figure S2

Temporal profiles of the enhancer activities. *Top line*, CNEs in the reporter constructs. *Second to bottom lines*, reporter GFP signals at 4, 7, 9, 12, 16, after 20 h after electroporation. *Left column* indicates the bright views of CMV-GFP electroporated embryo. *Scale bar*, 1 mm.

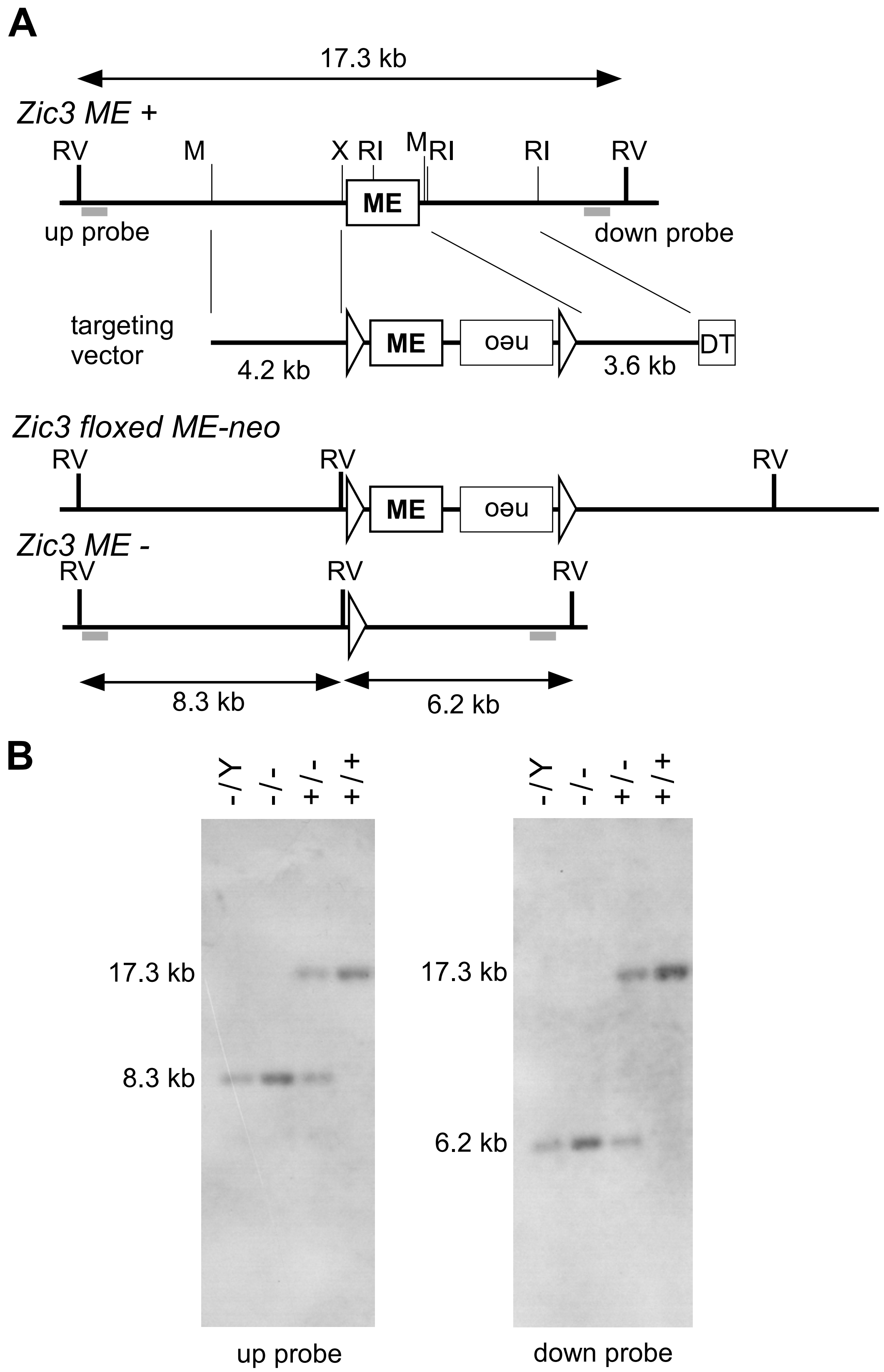

Supplementary Figure S3

Generation of Zic3-ME knockout mice

(A) Targeting strategy. The vector was designed for conditional knockout (*floxed Zic3-ME*). But conditional KO was not performed in this study. *RV*, EcoRV; *RI*, EcoRI; *M*, MscI; *X*, XhoI; *ME*, Zic3-ME; *triangles*, loxP sequence; *neo*, G418 resistant cassette; *DT*, diphtheria toxin A fragment.
(B) Results of Southern blot analysis after EcoRV digestion. *Top* indicates the genotype and *bottom* indicates the probes used for hybridization. The images derived from the same gel and the same blot. The blot was cut into two pieces, which were then hybridized with up or down probe independently and exposed to a film simultaneously.

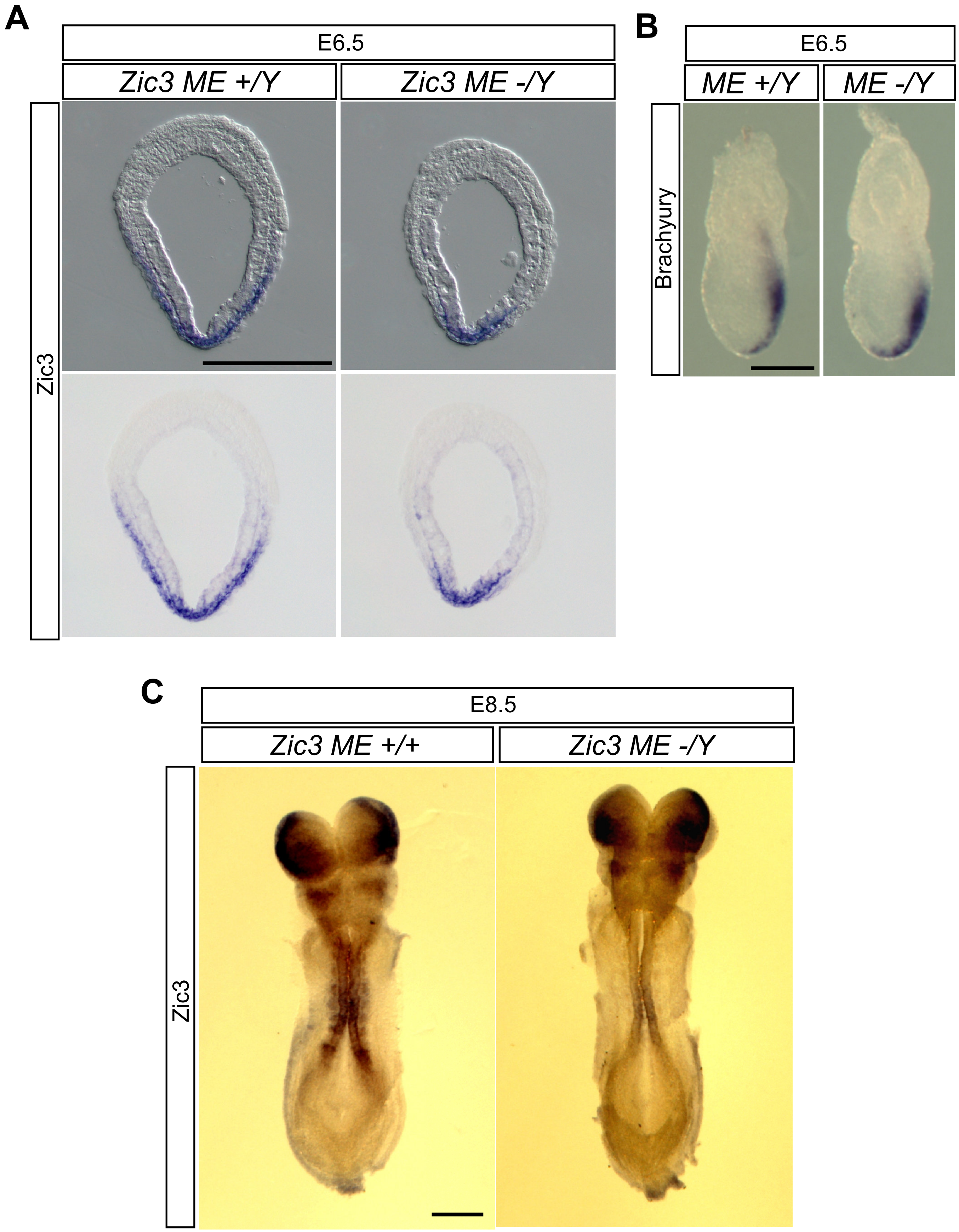

Supplementary Figure S4
Supplementary results for analysis of Zic3-ME-deficient mice (Fig. 4).
(A) Differential contrast images showing Zic3 transcript location in E6.5 embryo sections from wild-type (*Zic3 ME +/Y*) and Zic3-ME-deficient (*Zic3 ME-/Y*) (*Top*). Bright field views of the same sections are shown *below*. (B) Whole-mount *in situ* hybridization image showing Brachyury (Bra, T) transcript location in E6.5 wild-type (*ME +/Y*) and Zic3-ME-deficient (*ME-/Y*) embryos. (C) Zic3 transcript location in E8.5 (7 somite-stage) wild-type (*Zic3-ME*+/+) and Zic3-ME-deficient (*Zic3-ME-/Y*) embryos. *Scale bars*, 200 μm

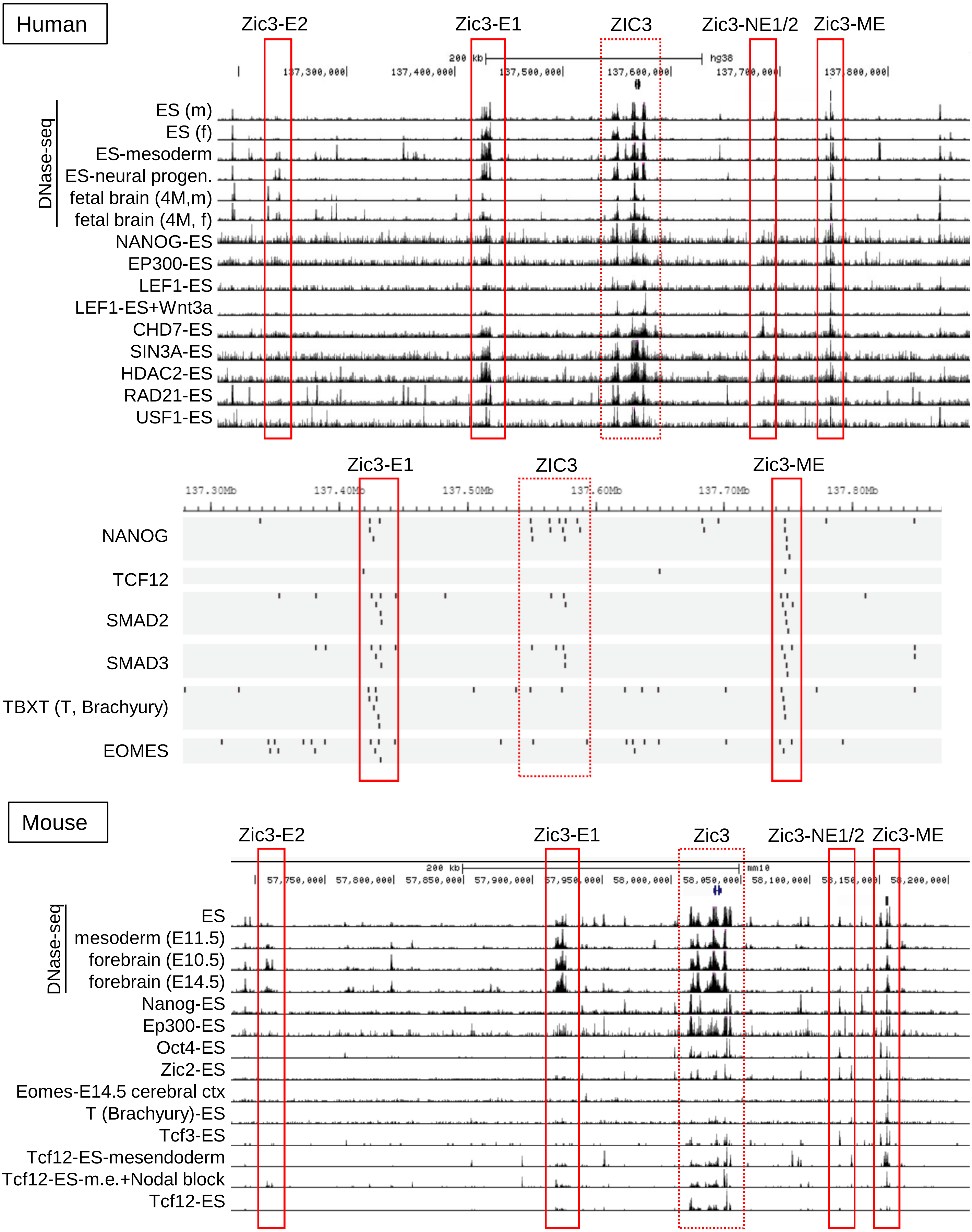

Supplementary Figure S5

Representative DNase-seq and ChIP-seq results including Zic3-enhancers in human and mouse. *Middle panel*, GTRD (ChIP-seq metapeaks, http://gtrd.biouml.org); *top and bottom panels*, ENCODE (htttps://www.encodeproject.org/) and CISTROME (http://Cistrome.org/db/#/). Higher magnification of Zic3-MEs are indicated in Fig. 6C. Derivation of the data is listed in Supplementary Table S1.

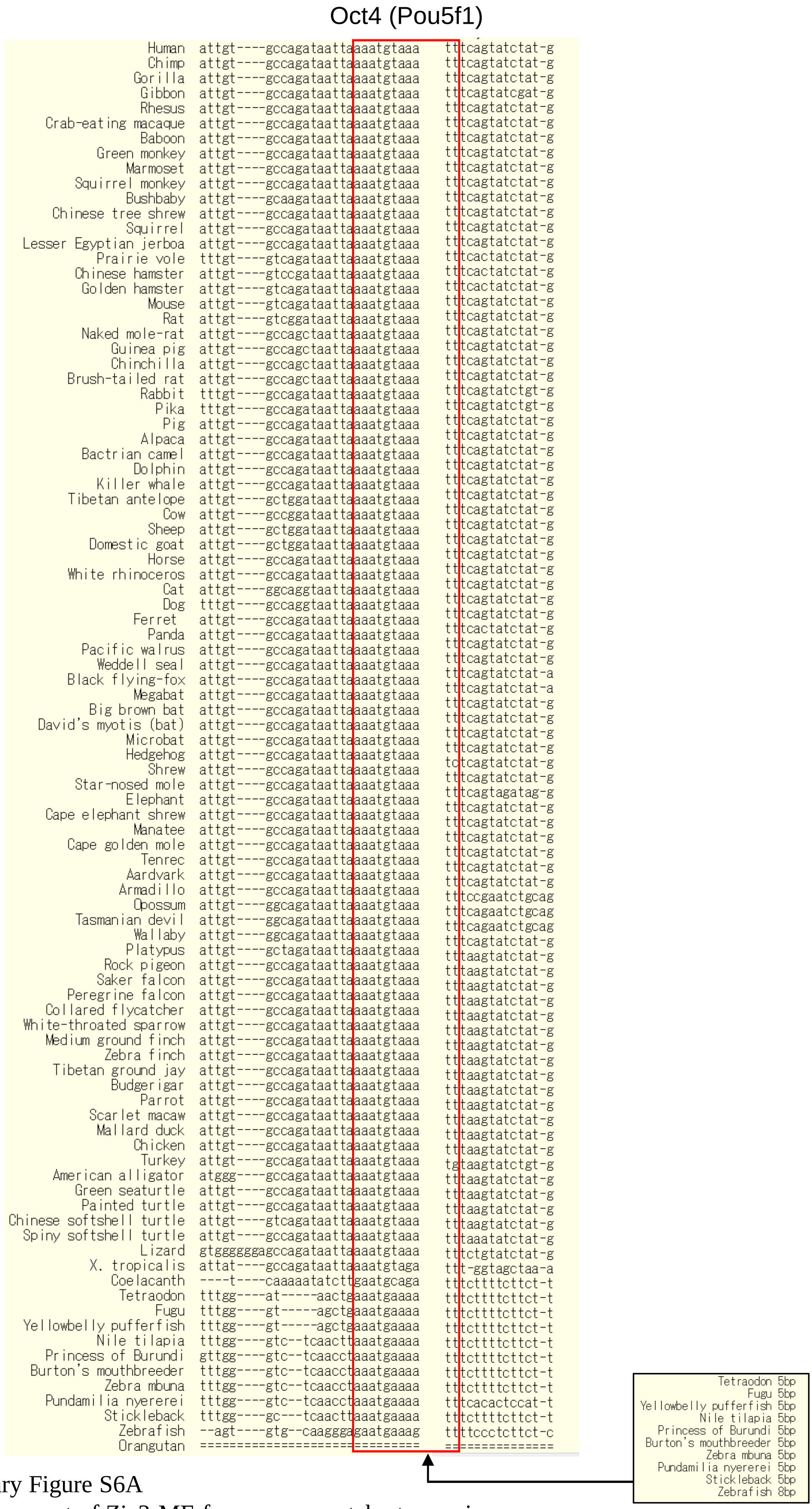

Supplementary Figure S6A
Sequence alignment of Zic3-ME from many vertebrate species.
(A-E) The alignment position corresponds to those indicated in Fig. 6B.
The alignment was obtained through UCSC genome browser.

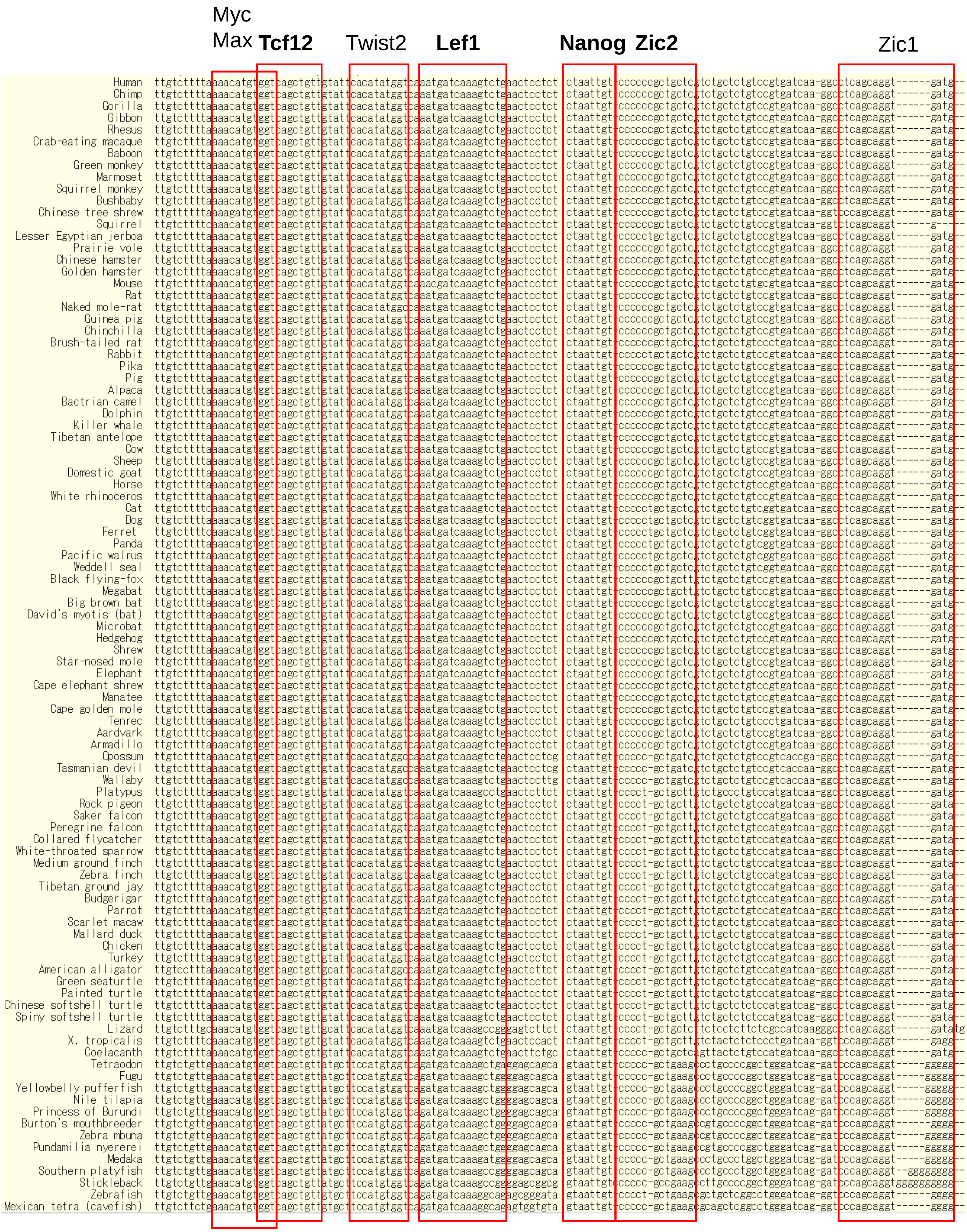

Supplementary Figure S6B

Oct4 (Pou5f1)

```
                    Human  aaatgcaaa-----------tgg
                    Chimp  aaatgcaaa-----------tgg
                  Gorilla  aaatgcaaa-----------tgg
                   Gibbon  aaatgcaaa-----------tgg
                   Rhesus  aaatgcaaa-----------tgg
       Crab-eating macaque  aaatgcaaa-----------tgg
                   Baboon  aaatgcaaa-----------tgg
             Green monkey  aaatgcaaa-----------tgg
                 Marmoset  aaatgcaaa-----------tgg
          Squirrel monkey  aaatgcaaa-----------tgg
                 Bushbaby  aaatgcaaa-----------tgg
       Chinese tree shrew  aaatgcaaa-----------tgg
                 Squirrel  aaatgcaaa-----------tgg
   Lesser Egyptian jerboa  aaatgcaaa-----------tgg
             Prairie vole  aaatgcaaa-----------tgg
          Chinese hamster  aaatgcaaa-----------tgg
           Golden hamster  aaatgcaaa-----------tgg
                    Mouse  aaatgcaaa-----------tgg
                      Rat  aaatgcaaa-----------tgg
           Naked mole-rat  aaatgcaaa-----------tgg
               Guinea pig  aaatgcaaa-----------tgg
               Chinchilla  aaatgcaaa-----------tgg
        Brush-tailed rat  aaatgcaaa-----------tgg
                   Rabbit  caatgcaaa-----------tgg
                     Pika  caactcaaa-----------tgg
                      Pig  aaatgcaaa-----------tgg
                   Alpaca  aaatgcaaa-----------tgg
           Bactrian camel  aaatgcaaa-----------tgg
                  Dolphin  aaatgcaaa-----------tgg
             Killer whale  aaatgcaaa-----------tgg
         Tibetan antelope  aaatgcaaa-----------tgg
                      Cow  aaatgcaaa-----------tgg
                    Sheep  aaatgcaaa-----------tgg
            Domestic goat  aaatgcaaa-----------tgg
                    Horse  aaatgcaaa-----------tgg
         White rhinoceros  aaatgcaaa-----------tgg
                      Cat  aaatggaag-----------tgg
                      Dog  caatgtaaa-----------tgg
                   Ferret  ccatgtaaa-----------tgg
                    Panda  ccatctaaa-----------tgg
           Pacific walrus  cagtgtaaa-----------tgg
             Weddell seal  caatgtaaa-----------tgg
        Black flying-fox  aaatgcaaa-----------tgg
                  Megabat  aaatgcaaa-----------tgg
            Big brown bat  aaatgcaaa-----------tgg
    David's myotis (bat)  aaatgcaaa-----------tgg
                 Microbat  aaatgcaaa-----------tgg
                 Hedgehog  aaatgcaaa-----------tgg
                    Shrew  aaatgcaaa-----------tgg
          Star-nosed mole  aaatgcaaa-----------tgg
                 Elephant  aaatgcaaa-----------tgg
      Cape elephant shrew  cagtgcaaa-----------tgg
                  Manatee  agatgcaaa-----------tgg
         Cape golden mole  aaatgcaaa-----------tgg
                   Tenrec  aaatgcaaa-----------tgg
                 Aardvark  aaatgcaaa-----------tgg
                Armadillo  aaatgcaaa-----------tgg
                  Opossum  gaatgcaaa-----------tgc
          Tasmanian devil  gaatgcaaa-----------tgt
                  Wallaby  gaatgcaaa-----------tgt
                 Platypus  aaatgcaaatgttggggggttgg
              Rock pigeon  aaatgcaaa-----------tgt
             Saker falcon  aaatgcaaa-----------tgg
         Peregrine falcon  aaatgcaaa-----------tgg
      Collared flycatcher  gaatgcaaa-----------tgg
  White-throated sparrow  gaatgcaaa-----------tag
      Medium ground finch  gaatgcaaa-----------tag
              Zebra finch  gaatgcaaa-----------tag
       Tibetan ground jay  gaatgcaaa-----------tag
               Budgerigar  gaatgcaaa-----------tgg
                   Parrot  gaatgcaaa-----------tgg
            Scarlet macaw  gaatgcaaa-----------tgg
             Mallard duck  aaatgcaaa-----------ttt
                  Chicken  aaatgcaaa-----------tgt
                   Turkey  aaatgcaaa-----------tgc
       American alligator  aaatgcaaa-----------tgc
          Green seaturtle  aaatgcaaa-----------tgt
           Painted turtle  aaatgcaaa-----------tgc
 Chinese softshell turtle  aaatgcaaa-----------ggc
   Spiny softshell turtle  aaatgcaaa-----------ggc
           X. tropicalis  acacataaa-----------t--
               Coelacanth  aaat-------------------
Mexican tetra (cavefish)  aaacacaga-----------tac
                   Lizard  =======================
                Orangutan  =======================
                Zebrafish  =======================
              Spotted gar =======================
```

Supplementary Figure S6C

T (TBXT, Brachyury)

```
                     Human  ----agttcactttgccagatgg--cg--ctttattgtcactagtggttacacct---cgctattggatc
                     Chimp  ----agttcactttgccagatgg--cg--ctttattgtcactagtggttacacct---cgctattggatc
                   Gorilla  ----agttcactttgccagatgg--cg--ctttattgtcactagtggttacacct---cgctattggatc
                    Gibbon  ----agttcactttgccagatgg--cg--ctttattgtcactagtggttacacct---cgctattggatc
                    Rhesus  ----agttcactttgccagatgg--cg--ctttattgtcactagtggttacacct---cgctattggatc
       Crab-eating macaque  ----agttcactttgccagatgg--cg--ctttattgtcactagtggttacacct---cgctattggatc
                    Baboon  ----agttcactttgccagatgg--cg--ctttattgtcactagtggttacacct---cgctattggatc
              Green monkey  ----agttcactttgccagatgg--cg--ctttattgtcactagtggttacacct---cgctattggatc
                  Marmoset  ----agttcactttgccagatgg--cg--ctttattgtcactagtggttacacct---cgctattggatc
           Squirrel monkey  ----agttcactttgccagatgg--cg--ctttattgtcactagtggttacacct---cgctattggatc
                  Bushbaby  ----agttcactttgccagatgg--cg--ctttattgtcactggtggttacacct---cgctattggatc
        Chinese tree shrew  ----agttcactttgccagatgg--cg--ctttattgtcactagtggttacacct---cgctattggatc
                  Squirrel  ----agttcactttgccagatgg--cg--ctttattgtcactagtggttacacct---cgctattggatc
     Lesser Egyptian jerboa  ----agttcactttgccagatgg--cg--ctttattgtcactagtggttacacct---cgctattggatc
              Prairie vole  ----agttcactttgccagatgg--cg--ctttattgtcactagtggttacacct---cgctattggatc
           Chinese hamster  ----agttcactttgccagatgg--cg--ctttattgtcactagtggttacacct---cgctattggatc
            Golden hamster  ----agttcactttgccagatgg--cg--ctttattgtcactagtggttacacct---cgctattggatc
                     Mouse  ----agttcactttgccagatgg--cg--ctttattgtcactagtggttacacct---cgctattggatc
                       Rat  ----agttcactttgccagatgg--cg--ctttattgtcactagtggttacacct---cgctattggatc
            Naked mole-rat  ----agttcactttgccagatgg--cg--ctttattgtcactggtggttacacct---cgctattggatc
                Guinea pig  ----agttcactttgccagatgg--cg--ctttattgtcactggtggttacacct---cgctattggatc
                Chinchilla  ----agttcactttgccagatgg--cg--ctttattgtcactggtggttacacct---cgctattggatc
          Brush-tailed rat  ----agttcactttgccagatgg--cg--ctttattgtcactggtggttacacct---cgctattggatc
                    Rabbit  ----agttcactttgccagatgg--cg--ctttattgtcactagtggttacacct---cgctattggatc
                      Pika  ----agttcactttgccagatgg--cg--ctttattgtcactagtggttacacct---cgctattggatc
                       Pig  ----agttcactttgccagatgg--cg--ctttattgtcactagtggttacacct---cgctattggatc
                    Alpaca  ----agttcactttgccagatgg--cg--ctttattgtcactagtggttacacct---cgctattggatc
            Bactrian camel  ----agttcactttgccagatgg--cg--ctttattgtcactagtggttacacct---cgctattggatc
                   Dolphin  ----agttcactttgccagatgg--cg--ctttattgtcactagtggttacacct---cgctattggatc
              Killer whale  ----agttcactttgccagatgg--tg--ctttattgtcactagtggttacacct---cgctattggatc
          Tibetan antelope  ----agttcactttgccagatgg--cg--ctttattgtcactagtggttacacct---cgctattggatc
                       Cow  ----agttcactttgccagatgg--cg--ctttattgtcactagtggttacacct---cgctattggatc
                     Sheep  ----agttcactttgccagatgg--cg--ctttattgtcactagtggttacacct---cgctattggatc
             Domestic goat  ----agttcactttgccagatgg--cg--ctttattgtcactagtggttacacct---cgctattggatc
                     Horse  ----agttcactttgccagatgg--cg--ctttattgtcactagtggttacacct---cgctattggatc
          White rhinoceros  ----agttcactttgccagatgg--cg--ctttattgtcactagtggttacacct---cgctattggatc
                       Cat  ----agttcactttgccagatgg--cg--ctttattgtcactggtggttacacct---cgctattggatc
                       Dog  ----agttcactttgccagatgg--cg--ctttattgtcactagtggttacacct---cgctattggatc
                    Ferret  ----agttcactttgccagatgg--cg--ttttattgtcaccactggttacacct---cgctattggatc
                     Panda  ----agttcactttgccagatgg--cg--ctttattgccactagtggttacacct---cgctattggatc
            Pacific walrus  ----agttcactttgccagatgg--cg--ctttattgtcactagtggttacacct---cgctattggatc
              Weddell seal  ----agttcactttgccagatgg--cg--ctttattgtcactagtggttacacct---cgctattggatc
          Black flying-fox  ----agttcactttgccagatgg--tg--ctttattgtcaccagtggttacacct---cgctattggatc
                   Megabat  ----agttcactttgccagatgg--tg--ctttattgtcaccagtggttacacct---cgctattggatc
             Big brown bat  ----agttcactttgccagatgg--cg--ctttattgtcactagtggttacacct---cgctattggatc
       David's myotis (bat)  ----agttcactttgccagatgg--cg--ctttattgtcactagtggttacacct---cgctattggatc
                  Microbat  ----agttcactttgccagatgg--cg--ctttattgtcactagtggttacacct---cgctattggatc
                  Hedgehog  ----agttcactttgccagatgg--cg--ctttattgtcactagtggttacacct---cgctattggatc
                     Shrew  ----agttcactttgccagatgg--cg--ctttattgtcactagtggttacacct---cgctattggatc
           Star-nosed mole  ----agttcactttgccagatgg--cg--ctttattgtcactagtggttacacct---cgctattggatc
                  Elephant  ----agttcactttgccagatgg--tg--ctttattgtcactagtggttacacct---cgctattggatc
       Cape elephant shrew  ----agttcactttgccagatgg--cg--ctttattgtcactagtggttacacct---cgctattggatc
                   Manatee  ----agttcactttgccagatgg--cg--ctttattgtcactagtggttacacct---cgctattggatc
           Cape golden mole  ----agttcactttgccagatgg--cg--ctttattgtcactagtggttacacct---cgctattggatc
                    Tenrec  ----agttcactttgccagatgg--cg--ctttattgtcactagtggtgacacct---cgctattggatc
                  Aardvark  ----agttcactttgccagatgg--cg--ctttattgtcactagtggttacacct---cgctattggatc
                 Armadillo  ----agttcactttgccagatgg--cg--ctttattgtcactagtggttacacct---cgctattggatc
                   Opossum  ----agttcactttaccagatgg--ct--ctgccttgtccccagcggctacacctccccaccattggagc
           Tasmanian devil  ----ggttcactttaccagatgg--ct--ctgccttgtccccagcagctacacct-cccgccactggagc
                   Wallaby  ----agttcactttaccagatgg--ct--ctgccttgtccctagcggctacacct-cccgccattggagc
                  Platypus  ----agttcactttgccagatgg--ct--ctttattgtcactagggtctacacct---ccctattggatc
               Rock pigeon  ----agttcactttgccagatgg--tt--ctttattgtcactggtgtccacacct---cgctcttgcatc
              Saker falcon  ----agttcactttgccagatgg--tt--ctttattgtcactggtgtccacacct---cactcttgcatc
          Peregrine falcon  ----agttcactttgccagatgg--tt--ctttattgtcactggtgtccacacct---cactcttgcatc
       Collared flycatcher  ----agctcactttgccagatgg--tg--ctttattgtcactggtgtccacacct---cgctcttgcatc
    White-throated sparrow  ----agttcactttgccagatgg--tg--ctttattgtcactggtgtccacacct---cactcttgcatc
       Medium ground finch  ----agttcactttgccagatgg--tg--ctttattgtcactggtgtccacacct---cactcttgcatc
               Zebra finch  ----agttcactttgccagatgg--tg--ctttattgtcactggtgtccacacct---cactcttgcatc
        Tibetan ground jay  ----agttcactttgccagatgg--tg--ctttattgtcactggtgtccacacct---cgctcttgcatc
                Budgerigar  ----agttcactttgccagatgg--tt--ctttattgtcactggtgtccacacct---cgctcttgcatc
                    Parrot  ----agttcactttgccagatgg--tt--ctttattgtcactggtgtccacacct---cgctcttgcatc
              Scarlet macaw  ----agttcactttgccagatgg--tt--ctttattgtcactggtgtccacacct---cgctcttgcatc
              Mallard duck  ----agttcactttgccagatgg--tt--ctttattgtcactggtgtccacacct---cgctcttgcatc
                   Chicken  ----agttcactttgccagatgg--tt--ctttattgtcactggtgtccacacct---cgctcttgcatc
                    Turkey  ----agttcactttgccagatgg--tt--ctttattgtcactggtgtccacacct---cgctcttgcatc
        American alligator  ----agttcacttggccagatggcctc--ttttattgtcacgagtgtccacacct---cgctattggatc
           Green seaturtle  ----agttcactttgccagatgg--ct--ctttattgtcactagtgtccacacct---cgcgattggatc
            Painted turtle  ----agttcactttgccagatgg--ct--ctttattgtcactagtgtccacacct---cgcgattggatc
  Chinese softshell turtle  ----agttcactttgccagatgg--ct--ctttattgtcactagtgtccacacct---cgtgattggatc
    Spiny softshell turtle  ----agttcactttgccagatgg--ct--ctttattgtcactagtgtccacacct---cgtgattggatc
                    Lizard  ----agc-cactttgccagatgg--ctactttattgtcgcccgtgtttacacct---cccgattggatc
            X. tropicalis  ----agttaactctgccagatgg--ct--ccttattgtcattggtttgtacacct---cactataggatc
                Coelacanth  ----agttcactttgccagatgg--tt--cattattatagctggatttacactt---ttctatccaata
               Spotted gar  cactgccccctctgccagatgatttt--ctttcctatc---------tgcccct---cagcgctgggtc
                 Orangutan  ======================================================================
                 Zebrafish  ======================================================================
Mexican tetra (cavefish)  =====================================================================
```

Supplementary Figure S6D

Eomes

```
                     Human  agttcaagaccaaatggtgtg--------aagagctttcatacttagaggatttagtcaaa---------
                     Chimp  agttcaagaccaaatggtgtg--------aagagctttcatacttagaggatttagtcaaa---------
                   Gorilla  agttcaagaccaaatggtgtg--------aagagctttcatacttagaggatttagtcaaa---------
                    Gibbon  agttcaagaccaaatggtgtg--------aagagctttcatacttagaggatttagtcaaa---------
                    Rhesus  agttcaagaccaaatggtgtg--------aagagctttcatacttagaggatttagtcaaa---------
        Crab-eating macaque  agttcaagaccaaatggtgtg--------aagagctttcatacttagaggatttagtcaaa---------
                    Baboon  agttcaagaccaaatggtgtg--------aagagctttcatacttagaggatttagtcaaa---------
              Green monkey  agttcaagaccaaatggtgtg--------aagagctttcatacttagaggatttactcaaa---------
                  Marmoset  agttcaagaccaaatggtgtg--------aagagctttcatacttagaggatttagtcaaa---------
            Squirrel monkey  agttcaagaccaaatggtgtg--------aagagctttcatacttagaggatttagtcaaa---------
                  Bushbaby  agttcaagaccaaatggtgtg--------aagagctttcatacttagaggatttagtcaaa---------
         Chinese tree shrew  agttcaagaccaaatggtgtg--------aagagctttcatacttagaggatttagtcaaa---------
                  Squirrel  agttcaagaccaaatggtgtg--------aagagctttcatacttagaggatttagtcaaa---------
      Lesser Egyptian jerboa  agttcaagaccaaatggtgtg--------aagagctttcatacgtagaggatttagtcaaa---------
               Prairie vole  agttcaagaccaaatggtgtg--------aagagctttcatacttagaggatttagccaaa---------
            Chinese hamster  agttcaagaccaaatggtgtg--------cagagctttcatacttagaggatttagccaaa---------
             Golden hamster  agttcaagaccaaatggtgtg--------cagagctttcatacttagaggatttagccaaa---------
                     Mouse  agttcaagaccaaatggtgtg--------aagagctttcatacttagaggatttagccaaa---------
                       Rat  agttcaagaccaaatggtgtg--------aagagctttcatacttagaggatttagccaaa---------
             Naked mole-rat  agttcaagaccaaatggtgtg--------aagagctttcatacttagaggatttaggcaaa---------
                 Guinea pig  agttcaagaccaaatggtgtg--------aagagctttcatacttagaggatttaggcaaa---------
                Chinchilla  agttcaagaccaaatggtgtg--------aagagctttcatacttagaggatttaggcaaa---------
           Brush-tailed rat  agttcaagaccaaatggtgtg--------aagagctttcatacttagaggatttaggcaaa---------
                    Rabbit  agctcaagaccaaatggtgtg--------aagagctttcatacttagaggatttagtcaaa---------
                      Pika  agctcaagaccaaatggtgtg--------aagagctttcatacttagaggatttagtcaaa---------
                       Pig  agttcaagaccaaatggtgtg--------aagagctttcatacttagaggatttagtcaaa---------
                    Alpaca  agttcaagaccaaatggtgtg--------aagagctttcatacttagaggatttagtcaaa---------
             Bactrian camel  agttcaagaccaaatggtgtg--------aagagctttcatacttagaggatttagtcaaa---------
                   Dolphin  agttcaagaccaaatggtgtg--------aagagctttcatacttagaggatttagtcaaa---------
              Killer whale  agttcaagaccaaatggtgtg--------aagagctttcatacttagaggatttagtcaaa---------
           Tibetan antelope  agttcaagaccaaatggtgtg--------aagagctttcatacttagaggatttagtcaaa---------
                       Cow  agttcaagaccaaatggtgtg--------aagagctttcatacttagaggatttagtcaaa---------
                     Sheep  agttcaagaccaaatggtgtg--------aagagctttcatacttagaggatttagtcaaa---------
             Domestic goat  agttcaagaccaaatggtgtg--------aagagctttcatacttagaggatttagtcaaa---------
                     Horse  agttcaagaccaaatggtgtg--------aagagctttcatacttagaggatttagtccaa---------
           White rhinoceros  agttcaagaccaaatggtgtg--------aagagctttcgtacttagaggatttagtcaaa---------
                       Cat  agttcgagaccaaatggtgtg--------aagagctttcatacttagaggatttagtcaaa---------
                       Dog  agttcaagaccaaatggtgtg--------aagagctttcatacttagaggatttagtcaaa---------
                    Ferret  agttcaagaccaaatggtgtg--------aagagctttcatactcagaggatttagtcgaa---------
                     Panda  agttcgagaccaaatggtgtg--------aagagctttcatacttagaggatttagtcaaa---------
             Pacific walrus  agttcaagaccaaatggtgtg--------aagagctttcatacttagaggatttagtcaaa---------
               Weddell seal  agttcaagaccaaatggtgtg--------aagagctttcatacttagaggatttagtcaaa---------
           Black flying-fox  agttcgagaccaaatggtgtg--------aagagctttcatacttagaggatttagtcaaa---------
                   Megabat  agttcgagaccaaatggtgtg--------aagagctttcatacttagaggatttagtcaaa---------
              Big brown bat  ggttcaagaccaaatggtgtg--------aagagctttcatacatagagggtttagtcaaa---------
        David's myotis (bat)  ggttcaagaccaaatggtgtg--------aagagctttcatacgtggagggtttagtcaaa---------
                  Microbat  ggttcaagaccaaatggtgtg--------aagagctttcatacgtagagggtttagtcaaa---------
                  Hedgehog  agttcaagaccaaatggtgtg--------aagagctttcatacttagaggatttagtcaaa---------
                     Shrew  agttcaagaccaaatggtgtg--------aagagctttcatacttagaggatttagtcaaa---------
            Star-nosed mole  agttcaagaccaaatggtgtg--------aagagctttcatacttagaggatttagtcaaa---------
                  Elephant  agttcaagaccaaatggtgtg--------aagagctttcatacttagaggatttagtcaaa---------
        Cape elephant shrew  agttcaagaccaaatggtgtg--------aagagctttcatacttagaggatttagtcaaa---------
                   Manatee  agttcaagaccaaatggtgtg--------aagagctttcatacttagaggatttagtcaaa---------
           Cape golden mole  agttcaagaccaaatggtgtg--------aagagctttcatacttagaggatttagtcaaa---------
                    Tenrec  agttcaagaccaaatggtgtg--------aagagctttcatacttagaggatttagccaaa---------
                  Aardvark  agttcaagaccaaatggtgtg--------aagagctttcatacttagaggatttagccaaa---------
                 Armadillo  agttcaagaccaaatggtgtg--------aagagctttcatacttagaggatttagtcaaa---------
                   Opossum  agttcaagaccaaatggtata--------gaaggctcttagggctggggattcaggagaa---------
            Tasmanian devil  agttcaagaccaaatggtgtg--------gagggcttttagggccaggggatttaggagaa---------
                   Wallaby  agttcaagaccaaatggtgtg--------gagggcttttagggtcaggggattt---tgaa---------
                  Platypus  agttcaagaccaaatggtgtg--------aagagctttaatacttaaaggatttagtaaaagtaaatcca
                Rock pigeon  acttcaagaccaaatggtgtg--------aagagctttaatacttaaaggatttagtaaaa---------
               Saker falcon  acttcaagaccaaatggtgtg--------aagagctttaatacttagaggatttagtaaaa---------
           Peregrine falcon  acttcaagaccaaatggtgtg--------aagagctttaatacttagaggatttagtaaaa---------
        Collared flycatcher  acttcaagaccaaatggtgtg--------aagagctttaatacttagaggatttagtaaaa---------
     White-throated sparrow  acttcaagaccaaatggtgtg--------aagagctttaatacttagaggatttagtaaaa---------
        Medium ground finch  acttcaagaccaaatggtgtg--------aagagccttaatacttagaggatttagtaaaa---------
               Zebra finch  acttcaagaccaaatggtgtg--------aagagctttaatacttagaggatttagtaaaa---------
         Tibetan ground jay  acttcaagaccaaatggtgtg--------aagagctttaatacttagaggatttagtaaaa---------
                Budgerigar  acttcaagaccaaatggtgtg--------aagagctttaatacttagaggatttagtaaaa---------
                    Parrot  acttcaagaccaaatggtgtg--------aagggctttaatacttagaggatttagtaaaa---------
              Scarlet macaw  acttcaagaccaaatggtgtg--------aagagctttaatacttagaggatttagtaaaa---------
               Mallard duck  acttcaagaccaaatggtgtg--------aagagctttaatacttagaggatttagtaaaa---------
                   Chicken  gcttcaagaccaaatggtgtg--------aagagctttaatacttagaggatttagtaaaa---------
                    Turkey  gcttcaagaccaaatggtgtg--------aagagctttaatacttagaggatttagtaaaa---------
         American alligator  gctgcgagaccaaatggtgtg--------aagagctttaagacttagaggatttaggaaaa---------
           Green seaturtle  acttcaagaccaaatggtgtg--------aagagctttaatactgagaggatttagtaaaa---------
            Painted turtle  acttcaagaccaaatggtgtg--------aagagctttaatactgagaggatttagtaaaa---------
   Chinese softshell turtle  actccaagaccaaatggtgtg--------aagagctttaatactgagaggatttagtaaaa---------
     Spiny softshell turtle  actccaagaccaaatggtgtg--------aagagctttaatactgagaggatttagtaaaa---------
                    Lizard  aagccaagaccaaatggtgtg--------aag---------acttagaagggtt--taagg---------
             X. tropicalis  agttcaagaccaaatggtgtg--------aaaagtgttaacgcttaaaggatttaattcaa---------
                Coelacanth  tgttcatggaaaaagggcatgcttaaaaaaagattttagaatccaaagtaattggaaga---------
                 Orangutan  ======================================================================
                 Zebrafish  ======================================================================
 Mexican tetra (cavefish)  =====================================================================
               Spotted gar  =====================================================================
```

Supplementary Figure S6E

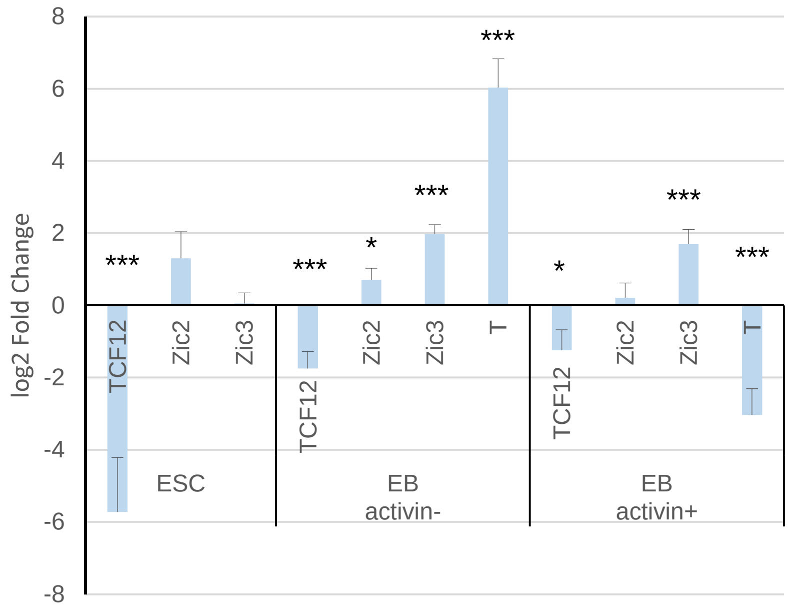

Supplementary Figure S7
Effects of Tcf12 knockdown on Zic3 expression.
Zic3 and reference genes (Tcf12, Zic2, and T) expression for biological triplicate shControl- and shTcf12-infected embryonic stem cells (ESC) and differentiated embryoid body (*EB*) in serum-free defined culture system with or without activin treatment. *ESC*, Mouse ESC CGR8; *EB/Activin-*, No treatment of Activin; *EB/Activin+*, At day 2, human Activin A (100 ng/ml) was added to induce endoderm differentiation without dissociation/reaggregation. Log2 fold change in comparison to the shControl transfected ESCs. *Error bar*, SEM. *$P < 0.05$, **$P < 0.01$, ***$P < 0.001$ in Wald test implemented in DEseq2. The results derive from publicized RNA-seq data (GSE60285_DESeq2_analysis_ESC.tsv, GSE60285_DESeq2_analysis_EB_activin-.tsv, GSE60285_DESeq2_analysis_EB_activin+.tsv) [59].
